# An assessment and characterization of pharmaceuticals and personal care products (PPCPs) within the Great Lakes Basin: Mussel Watch Program (2013–2018)

**DOI:** 10.1007/s10661-023-12119-3

**Published:** 2024-03-05

**Authors:** Edwards M. A., Kimbrough K., Fuller N., Davenport E., Rider M., Freitag A., Regan S., Leight A, K., Burkart H., Jacob A., Johnson E.

**Affiliations:** 1 Monitoring and Assessment Branch, NOAA/NOS/NCCOS, 1305 East/West Highway, Silver Spring, MD 20910 USA; 2 CSS-Inc., Under NOAA National Centers for Coastal Ocean Science Contract No, EA133C17BA0062 & EA133C17BA0049, Fairfax, VA USA

**Keywords:** Great Lakes, PPCPs, Dreissenid mussels, Land-use, Point source, WWTPs

## Abstract

Defining the environmental occurrence and distribution of chemicals of emerging concern (CECs), including pharmaceuticals and personal care products (PPCPs) in coastal aquatic systems, is often difficult and complex. In this study, 70 compounds representing several classes of pharmaceuticals, including antibiotics, anti-inflammatories, insect repellant, antibacterial, antidepressants, chemotherapy drugs, and X-ray contrast media compounds, were found in dreissenid mussel (zebra/quagga; *Dreissena* spp.) tissue samples. Overall concentration and detection frequencies varied significantly among sampling locations, site land-use categories, and sites sampled proximate and downstream of point source discharge. Verapamil, triclocarban, etoposide, citalopram, diphenhydramine, sertraline, amitriptyline, and DEET (N,N-diethyl-meta-toluamide) comprised the most ubiquitous PPCPs (> 50%) detected in dreissenid mussels. Among those compounds quantified in mussel tissue, sertraline, metformin, methylprednisolone, hydrocortisone, 1,7-dimethylxanthine, theophylline, zidovudine, prednisone, clonidine, 2-hydroxy-ibuprofen, iopamidol, and melphalan were detected at concentrations up to 475 ng/g (wet weight). Antihypertensives, antibiotics, and antidepressants accounted for the majority of the compounds quantified in mussel tissue. The results showed that PPCPs quantified in dreissenid mussels are occurring as complex mixtures, with 4 to 28 compounds detected at one or more sampling locations. The magnitude and composition of PPCPs detected were highest for sites not influenced by either WWTP or CSO discharge (i.e., non-WWTPs), strongly supporting non-point sources as important drivers and pathways for PPCPs detected in this study. As these compounds are detected at inshore and offshore locations, the findings of this study indicate that their persistence and potential risks are largely unknown, thus warranting further assessment and prioritization of these emerging contaminants in the Great Lakes Basin.

**Graphical Abstract:**

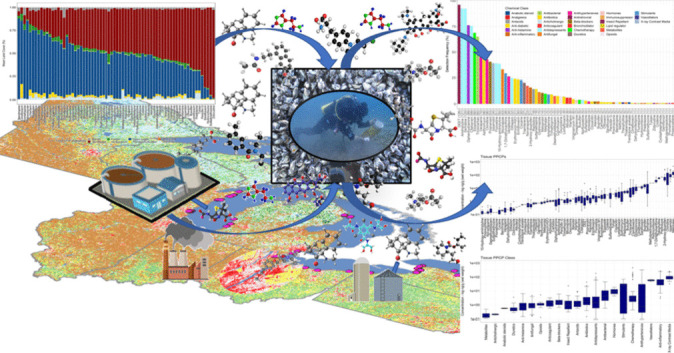

**Supplementary Information:**

The online version contains supplementary material available at 10.1007/s10661-023-12119-3.

## Introduction

Pharmaceuticals and personal care products (PPCPs) comprise a wide suite of prescription and over-the-counter (non-prescription medications) compounds intended for human and veterinary use, including animal production (e.g., livestock production and poultry processing), that are widely detected in aquatic and terrestrial environments (Ebele et al., 2017; Jaffrézic et al., 2017). Pharmaceutical products generally consist of biologically active organic compounds including but not limited to antibiotics, antidepressants, stimulants, X-ray contrast media drugs, anti-inflammatory, lipid regulators, β-blockers, antihypertensive compounds, and illicit/recreational drugs (Richmond et al., 2017; Yang, et al., 2017). Personal care products (PCPs) include chemical compounds found in household products, cosmetics, and health care products, such as sunscreen agents, antifungals, insect repellents, and antibacterial agents (i.e., triclosan and triclocarban; Celeiro et al., 2022). PPCPs are recognized as chemicals of emerging concern (CECs) due to their increased use in human and veterinary medicine, pseudo-persistence (e.g., widespread usage and continuous release/loading, resulting in measurable levels in the environment; Dehm et al., 2021), and frequent detection in industrial and municipal wastewater treatment systems, soil, sediment, drinking water, ground water, and surface water (Zakari-Jiya et al., 2022).

Typically, conventional wastewater treatment systems are designed to reduce or remove organic pollutants such as PPCPs, through secondary and tertiary/advanced treatment processes, including advanced oxidation processes (e.g., fenton, ozonation, and photolysis), membrane bioreactors (MBRs), and conventional activated sludge (CAS) treatment (Talib & Randhir, 2017). However, several studies have shown that wastewater treatment plant (WWTP) processes are unable to remove some of these pollutants during conventional wastewater treatment processes (Ebele et al., 2017; Liu et al., 2022; Rizzo et al., 2019). The partial elimination of PPCPs in conventional wastewater treatment allows for these contaminants to enter aquatic systems as parent compounds, metabolites, or transformation products (TP; Noguera-Oviedo & Aga, 2016; Pal et al., 2014; Wilkinson et al., 2017). Several studies have shown that most PPCPs are biorecalcitrant based on their physicochemical properties and stable structures, thus allowing their persistence in aquatic environments for extended periods, usually from weeks (e.g., oxazepam, iopromide, and ivermectin — 15–54 days) to months (e.g., diazepam and carbamazepine — 119–328 days; Ebele et al., 2016; Liu et al., 2022; Ren et al., 2021; Wang & Chu, 2016).

PPCP loading from point and diffuse sources including fugitive discharge from onsite medical wastewater, veterinary facilities, leaking septic systems, combined sewer overflow (CSOs), and discharge from sanitary treatment plants (STPs) continues to create a host of environmental challenges within the Great Lakes Basin (Hull et al., 2015; Jorgenson et al., 2018). More importantly, the environmental occurrence and distribution of contaminants in coastal freshwater systems are affected by coastal watershed characteristics such as increases in population density, urbanization, land cover fragmentation, and agricultural practices (Baldwin et al., 2016; Elliott, et al., 2017; Kiesling et al., 2019; Thomas et al., 2017). Additional studies have shown that PPCP detection and frequency can vary among coastal watersheds based on complex interactions between consumption and usage patterns, incomplete removal from conventional wastewater treatment processing, and proximity to point sources, thus making the occurrence, distribution, and potential sources of these contaminants in the Great Lakes difficult to identify and predict (Challis et al., 2018; Kiesling et al., 2019).

In addition to conventional wastewater discharge, episodic pollutant release and discharge from land-use gradients, including urban and agricultural runoff, can have direct impact on the occurrence and spatial distribution of organic pollutants in aquatic systems (Cantwell et al., 2018; Hwang et al., 2016). With agricultural, developed (combined developed open space, developed low intensity, developed medium intensity, and developed high intensity land-use categories; Homer et al., 2020), and urban land uses among some of the main land-use categories in the Great Lakes, runoff and anthropogenic pressures associated with these gradients have been closely linked to a series of environmental and water quality issues (Baldwin et al., 2016; Choy et al., 2017). As urban clusters expand, land-use gradients within these clusters may also change in terms of increased impervious surfaces, which results in increased non-point/diffuse source runoff (Shi et al., 2017). Moreover, increased anthropogenic pressures, as well as pollutant runoff from land-use based activities, including agricultural application of sludge and dewatered biosolids, have led to complex mixtures of organic pollutants, including PPCPs, to enter coastal freshwater environments and riverine systems (Wolter et al., 2006; Wu et al., 2009). Once these contaminants enter coastal aquatic systems, their exposure, environmental occurrence, and spatial distribution can become more complex and less understood (Wilkinson et al., 2017). Thus, continued monitoring and assessment is needed to examine and differentiate PPCPs that are uniquely generated from distinct land-use gradients, as well as those from point and diffuse sources.

One successful model for monitoring organic contaminants in the aquatic environment involves sampling filter-feeding bivalve species for contaminant loads. Since 1992, the National Oceanic and Atmospheric Administration (NOAA) Mussel Watch Program (MWP) has used dreissenid mussels to monitor the environmental occurrence and magnitude of a wide suite of organic pollutants in the Laurentian Great Lakes, establishing one of the most spatially robust biomonitoring data sets in the region (Edwards et al., 2016; Kimbrough et al., 2018). To date, the MWP has sampled over 300 locations across the Great Lakes Basin, including most major rivers and all five of the Great Lakes. By augmenting its basin-wide assessment with the use of caged mussels, the MWP expanded its monitoring efforts in the Great Lakes to include addressing place-based assessments of non-targeted contaminants, including PPCPs in dreissenid mussels (Kimbrough et al., 2018). Dreissenid mussels are extensively used as bioindicator organisms due to their unique attributes of high population density (≥ 700,000 specimens/${\mathrm{m}}^{2}$; Carvalho et al., 2021), sessile and sedentary nature, wide geographic distribution, quick response to contamination changes in aquatic systems, and notable longevity (3 to 5 years) (González-Fernández et al., 2015; James et al., 2020). Other characteristics which enable dreissenid mussels as excellent bioindicators include their ability to sequester and bioaccumulate contaminants from water and suspended sediments, thus reflecting ambient contamination levels in surrounding aquatic systems (Bruner et al., 1994; Kimbrough et al., 2014).

Summarizing all MWP tissue CEC data collected from dreissenid mussel surveillance and temporal sampling, basin-wide retrospective analyses, priority contaminant mixtures (PCM) studies, place-based contaminant assessment, and integrated assessment case studies (IACS) in the Great Lakes between 2013 and 2018, we conducted a post hoc analysis to (1) assess and characterize the environmental occurrence, magnitude, and spatial distribution of PPCPs detected in dreissenid mussels sampled at inshore (harbors, rivers, embayment, tributaries), and offshore locations; (2) examine potential drivers and gradients behind the occurrence and distribution of PPCPs detected at MWP sites; (3) explore the relationship between land-use patterns and PPCP concentrations detected in dreissenid mussels; and (4) assess site proximal location to point source and along gradients of wastewater discharge effect on PPCP concentration and composition. By leveraging the 2013–2018 tissue CEC contaminant data, a series of multivariate and unsupervised statistical techniques, including cluster analysis and random forest (RF) classification techniques, were developed to examine and identify patterns in PPCP distribution and environmental occurrence in the Great Lakes. This information is important in developing integrated approaches for source identification and suitable mechanisms for identifying and prioritizing contaminants of interest and their likely “hotspots” within the Great Lakes Basin.

## Methods

### Study area

The Laurentian Great Lakes constitutes the largest freshwater system in the world, containing an estimated 21% of the Earth’s surface freshwater (~ 90% of US freshwater; Fields, 2005; Sponberg, 2009). The Great Lakes Basin covers a total area of 244,000 km^2^ (94,000 mi^2^) and is home to over 35 million people (Breffle et al., 2013; Danz et al., 2007). The region watersheds which drain almost 518,000 km^2^ (200,000 mi^2^) support numerous economic industries, including manufacturing, agriculture, and commercial fisheries, with areas of intense urbanization and industrialization occurring along its coastal zone (Breffle et al., 2013; Wolter et al., 2006). Land-use and land cover differ between eco-regions and eco-provinces, with predominantly forested areas in the northern and southeastern sections, and agricultural activities more pronounced in the western and central sections of the Basin (Morrice et al., 2008). Increased anthropogenic and environmental stressors, including rapid urban growth and municipal and industrial wastewater discharge, have led to increased water quality impairment within the Great Lakes Basin and adjacent sub-watersheds (Danz et al., 2007; Elliot et al., 2017; Kiesling et al., 2022). Specifically, the continuous loading of organic pollutants from point and diffuse/fugitive sources has been identified as an environmental driver behind coastal water quality and ecosystem impairment within the Great Lakes (Baldwin et al., 2016; Cornwell et al., 2015; Kiesling et al., 2019).

### Site designation and categorization

**Fig. 1:**
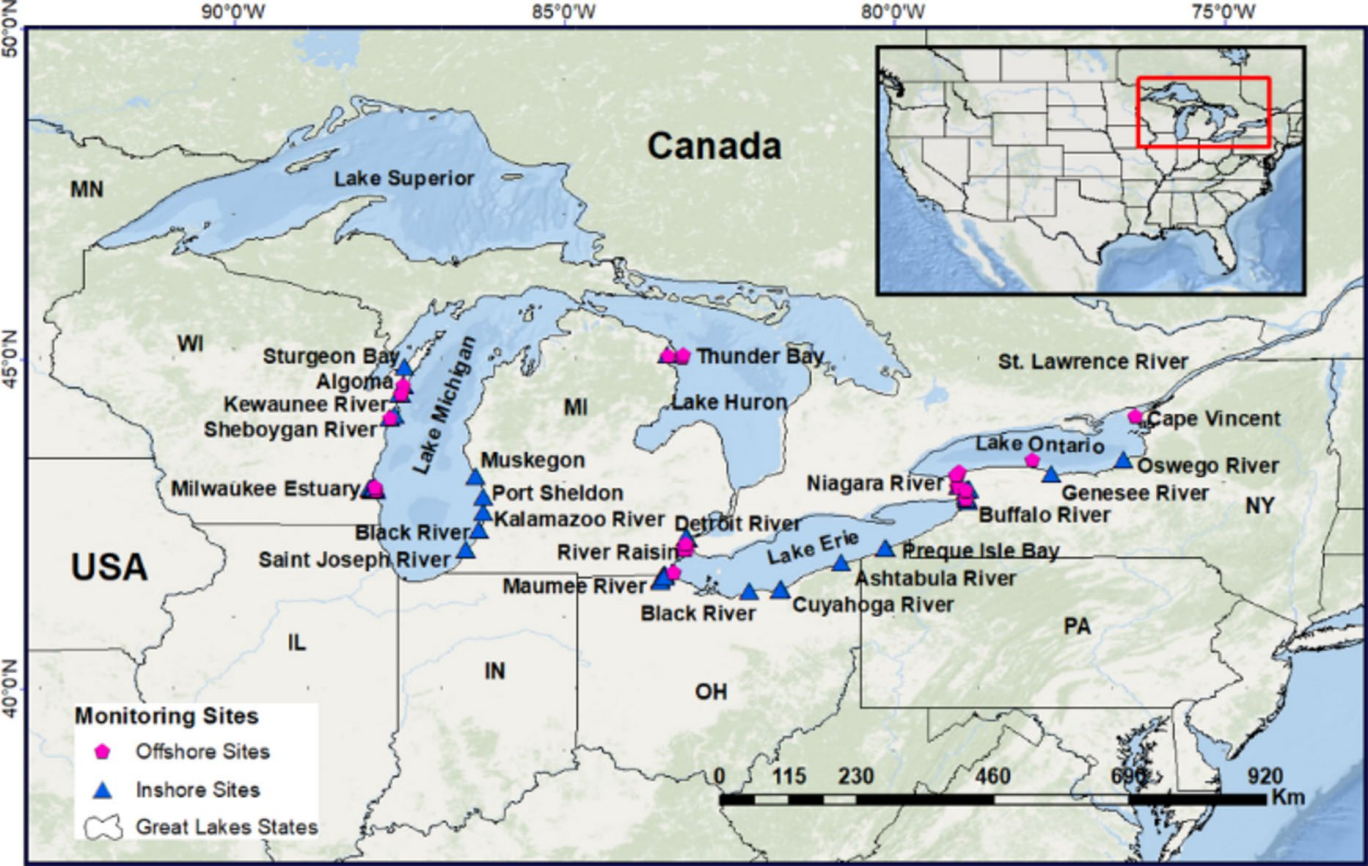
Great Lakes Mussel Watch Program (MWP) inshore (rivers, harbors, embayment, and tributaries) and offshore (nearshore lake and deep-water lake) dreissenid mussel 2013–2018 sampling locations. Some sampling locations have multiple sites. Most sampling locations have 1–3 sites, except Maumee River (8 sites), Muskegon (9 temporal sites), Milwaukee Estuary (13 sites), and Niagara River (20 sites). Additional information on MWP sampling locations description is provided in Table S1 and Table S10 (Supplementary Information)

A total of 131 sites, representing inshore and offshore locations in the Great Lakes Basin, were sampled between 2013 and 2018 (Fig. 1). A detailed description of the Great Lakes MWP study site locations including designated reference sites is provided in Table S1 (Supplementary Information) and described elsewhere (Edwards et al., 2016; Kimbrough et al., 2018, 2021). Additional information on MWP sampling locations is also provided in Table S10. Collectively, 14 sites were sampled in 2013, 29 sites were sampled in 2014, 15 sites were sampled in 2015, 12 sites were sampled in 2016, 19 sites were sampled in 2017, and 42 sites were sampled in 2018. Overall, CEC data from dreissenid mussel tissue were generated from multiple contamination assessment studies conducted under the expanded Great Lakes MWP basin-wide contaminant monitoring objectives. These objectives included addressing the Great Lakes Restoration Initiative (GLRI) Action Plan I (2010–2014) and GLRI Action Plan II (2015–2019; Kimbrough et al., 2018). To increase the likelihood of finding PPCPs, sites were preferentially selected based on the following: (1) riverine systems and tributaries known for higher contamination based on previous MWP studies, and (2) pollution gradients influenced by urban and sub-urban centers that receive high volume of pollutants from point source discharge and urban/storm-water runoff. MWP sites established in the Great Lakes Areas of Concern (AOC; areas designated for restoration due in part to historical environmental contamination; Hartig et al., 2020) and priority urban areas, including Milwaukee, Niagara, Toledo, Cleveland, and Detroit, were targeted with the objective of providing a more robust measure of bioavailable contaminants that would be generated from municipal, industrial, and fugitive sources, including urban storm water runoff (Kimbrough et al., 2021).

Multiple techniques including monitoring studies and place-based/caged mussel deployment were used to conduct contamination assessments and contaminant source tracking at inshore riverine and nearshore sites (Kimbrough et al., 2021). Mussels sampled at designated offshore locations included all lake sites (nearshore lake and deep-water lake sites), as well as sites sampled in the Great Lakes connecting channels. Inshore sampling locations include sites sampled at harbor, river, embayment, and tributary locations. Overall, most sampling locations have 1–3 sites, except for sampling locations in the Maumee River, Detroit River, Muskegon, Milwaukee Estuary, and Niagara River.

### Sampling procedures

Information on MWP sampling procedures is presented elsewhere (Kimbrough et al., 2018, 2021), as sampling methods and procedures were slightly different for various MWP basin-wide contaminant monitoring objectives. Sampling procedures conducted in this study utilized both in situ and caged dreissenid mussels for basin-wide surveillance monitoring and place-based contaminant assessment studies. When available, divers harvested in situ dreissenid mussels from established populations in open lake, near-shore lake zone, or from outer harbor stone breakwaters (Kimbrough et al., 2018). For sampling locations and place-based contaminant assessment sites where in situ mussels were unavailable, mussels were harvested by divers from nearshore harbor and stone breakwaters, placed in cages (e.g., minnow traps; approximately 300–500 mussels per cage), and deployed at selected sites for 28–55 days. For temporal studies, mussels were caged for up to 55 days.

The use of caged mussels in biomonitoring studies is well established. For example, studies have shown that caged mussels can be strategically deployed along known or suspected pollutant gradients to track and measure source contaminant exposure over time (de Solla et al., 2016; Guerlet et al., 2007; James et al., 2020; Salazar & Salazar, 1995). Additional studies have shown that caging mussels for 28–30 days (4 weeks) avoids bias relating to physiological and reproductive status that might influence organic pollutant uptake and bioaccumulation over the period of exposure (Tsangaris et al., 2010; Viarengo et al., 2007). Dreissenid mussels collected from in situ and place-based/caged deployment was rinsed with site water to remove debris, placed in labeled freezer bags, packed in ice containers, and shipped to contract laboratories within 2 days for analysis. Homogenates with more than 100 individuals were used for chemical analysis. Tissue sample collection and processing were consistent with NOAA methods and procedures for bivalve tissue assessment.

### Chemical analysis

Mussel tissue samples were analyzed at SGS AXYS Analytical Services (Sidney, British Columbia, Canada). A total of 141 contaminants were analyzed in mussel tissue samples (Table S2). Tissue samples were analyzed using a series of high-performance liquid chromatography reversed phase C18 or HILIC columns, combined with tandem mass spectrometry (HPLC/MS/MS) platform extraction, including gas chromatography coupled with high-resolution mass spectrometry (GC-HRMS) extraction. Individual PPCP analytes measured by positive or negative mode electrospray ionization (+ / − ESI) liquid chromatography tandem mass spectrometry (LC/MS/MS), or electron impact (EI) or electron capture negative chemical ionization (ECNI) mode gas chromatography mass spectrometry (GC/MS), were analyzed from modified methods described in EPA Method 1694 and 1698 (Dodder et al., 2014; U.S. EPA 2007). SGS AXYS quality assurance criteria used for method MLA-075 Rev 06.01 and method MLA-075 Rev 07 included analyses of laboratory blanks, duplicate samples, labeled quantification standard recovery, and blind field sample duplicates.

### Quality assurance and quality control

Standard SGS AXYS laboratory quality control measures used for mussel tissue analyses are described elsewhere (James et al., 2020). Briefly, mussel tissue analyses were performed in batches. Each batch consisted of test samples and additional QC samples, with ultrapure water used as the blank matrix. Procedural blanks were extracted and analyzed using the same procedures as the test samples in the analysis batch. Standard SGS AXYS laboratory quality control measures, which included matrix blanks and matrix spikes, were also used for mussel tissue analysis. Only blank corrected concentrations above the method detection limit were recorded as present. In addition, all PPCPs quantified in mussel tissue were prioritized and grouped into 27 classes, based on their primary therapeutic class, with concentration for each compound class summed to provide a cumulative total PPCP concentration measured at each MW site.

### Data analysis

#### Land-use and point source data

The land-use and land cover (LULC) assessment conducted in this study is described elsewhere (Edwards et al., 2014, 2016; Kimbrough et al., 2021; Freitag et al., 2021). A brief overview is provided here to describe additional techniques used to develop and determine LULC estimates for each Great Lakes MWP study site. Site LULC estimates were mainly created from the USGS Multi-Resolution Land Characteristics (MRLC) (https://www.mrlc.gov/data/nlcd-2016-land-cover-conus) and 2016 National Land Cover Database (NLCD) raster layer (Homer et al., 2020). However, due to differences in US and Canadian LULC spatial coverages, spatial data analyses were performed slightly differently depending on the location of MWP sites. Specifically, for sites near the Canadian portion of the Great Lakes Basin, LULC analyses involved merging and remapping US and Canadian LULC data (https://open.canada.ca/data/en/dataset/4e615eae-b90c-420b-adee-2ca35896caf6), based on the Anderson LULC values (Anderson et al., 1976; Choy et al., 2017; Yang et al., 2018).

**Fig. 2:**
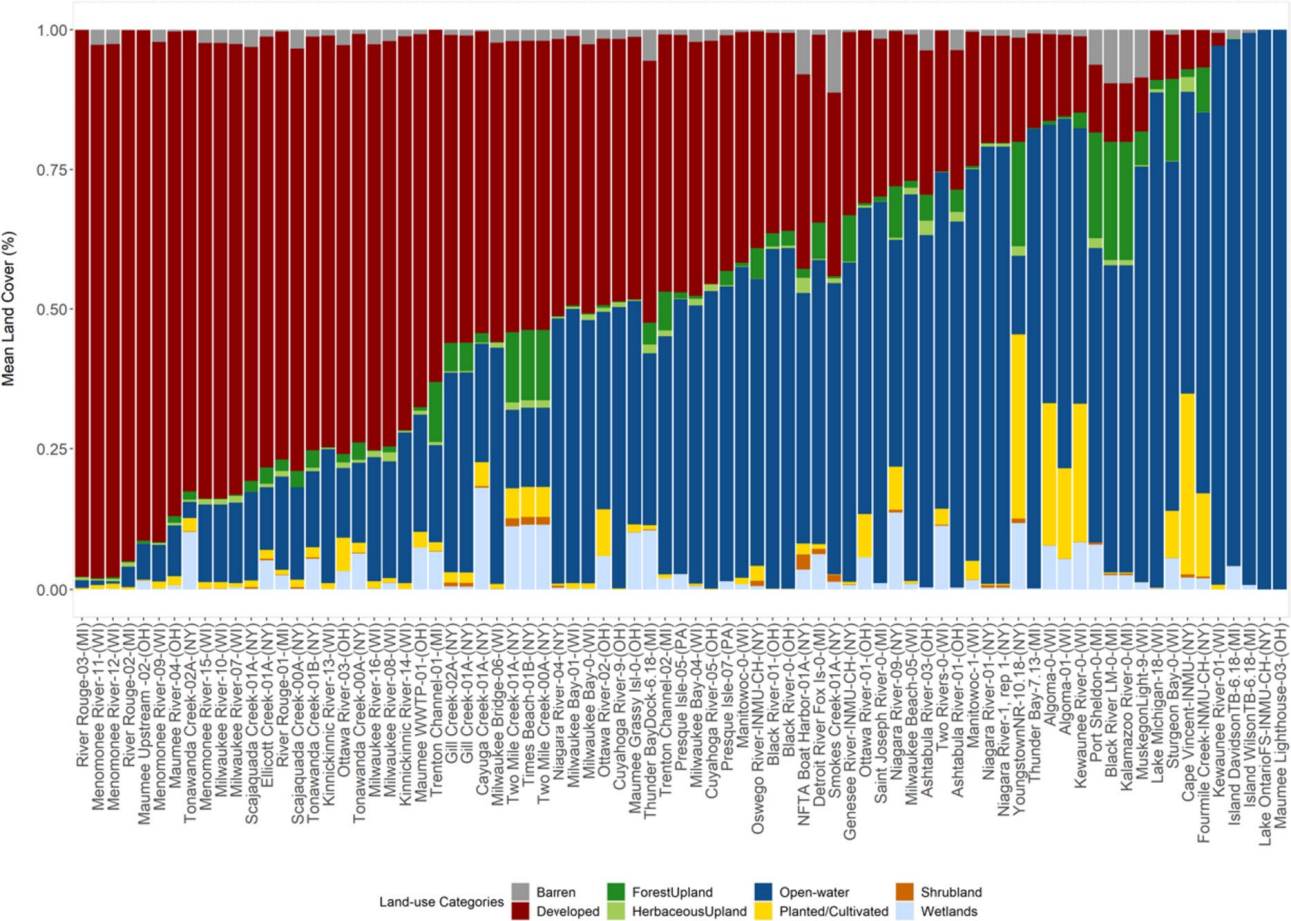
Great Lakes dreissenid mussel sampling location associated land-use categories and land cover estimates (%) found proximate (within 3-km buffer) to each MWP site. Sampling locations land cover estimates (%) are presented in order from greatest to least developed land cover category. Land-use information is presented for most MW sites, as some sites were removed due to space constraints. Table S10 (Supplementary Information) provides more details on the land-use categories and land-cover estimations at MWP sites. Individual sites are listed by their general location (associated river/lake region) and state which corresponds with mussel study sites provided in Table S1

A 3-km buffer radius was adopted, based on the distance between MWP sampling locations, and the influence of adjacent land-use and point/diffuse source chemical loading around MWP sites at designated Great Lakes inshore and offshore locations. For simplification purposes, the NLCD LULC classes were reclassified and aggregated into eight generic land-use categories (Developed, Planted/Cultivated, Shrubs, Barren, Herbaceous, Forest, Wetlands, and Open-water; Fig. 2; Table S3). Land cover estimates within each 3-km buffer were further used to sort MWP sites into exclusive categories, with developed and open-water being the dominant land-use categories (Table S10). Planted/cultivated land cover category was dominant at one study site (NRYT-INMU-CH-10.18), but was not included in the final assessment of PPCPs detected at mussel study sites, since there were not enough agricultural sites available for comparison and subsequent discussion. The lack of agricultural sites might pose some limitations, since previous studies have shown watersheds associated with agricultural land uses also influence PPCP loading in the Great Lakes (Cipoletti et al., 2020; Kiesling et al., 2019; Wu et al., 2009). However, the Great Lakes MWP CEC sites were specifically established in urban watersheds and associated land uses to better identify and address chemical contamination at inshore and offshore locations in the Great Lakes Basin (Kimbrough et al., 2018).

Digital maps and appropriate orthoimagery datasets (1:12,000-scale or greater from the National Agriculture Imagery Program (NAIP)) covering the period 2006 to 2013 were used to identify and clip appropriate US Environmental Protection Agency (EPA) National Pollution Discharge Elimination System (NPDES) major and minor permitted WWTPs, CSO shapefiles, and 2010 US Census Bureau population and demographic data at the block-group level within each study site 3-km buffer. WWTP effluent data were generated from existing International Joint Commission (IJC) information on Great Lakes WWTPs (Laitta, 2016) and the US EPA’s Enforcement and Compliance History Online (ECHO) facility program (ECHO, 2022). Study site location and proximity to individual USEPA non-permitted and permitted NPDES facilities were determined through Geographic Information System (GIS) analysis using ArcMap 10.8 software (ESRI, Inc.).

### Statistical analysis

An unsupervised machine learning approach that included random forest (RF) classification was used to find patterns in PPCP presence/absence data, as well as characterize the relationship between PPCP concentration and study sites land-use and land cover. RF classification techniques were conducted using the R statistical software (R version 4.0.2; RCore Team, 2020), with the “randomForest” package for unsupervised RF classification, followed by cluster analysis which was used to group the RF classification results into distinct clusters. The number of clusters was determined using the gap statistic and Mclust package (Fraley et al., 2012).

Statistical analyses were conducted using non-parametric Kruskal–Wallis tests, followed by pairwise comparisons, using Wilcoxon rank sum test and Dunn Kruskal–Wallis multiple comparison post hoc test (*p* < 0.05), with Benjamini-Hochberg (BH) method. Spearman’s (non-parametric) rank correlation (*ρ*) analysis was used to explore the relationship between PPCP compound groups and study site parameters, including site land-use categories, site population estimates, point sources (WWTP and CSOs), and WWTP flow. Spearman’s (non-parametric) rank correlation (*ρ*) analyses were also conducted using the R statistical software (R version 4.0.2; RCore Team, 2020). Finally, data summaries, including descriptive statistics for PPCP compounds measured in dreissenid mussel tissue, are provided and organized by RF clusters, sampling location (inshore and offshore), major discharge types, and land-use categories.

## Results and discussion

A summary of PPCP contaminants detected in this retrospective/post hoc study, based on the most recent dataset (2013–2018) to be found in dreissenid mussels from the Great Lakes, is presented here. Comprehensive measurements and assessment within river-tributary-harbor complexes, lake nearshore, and lake offshore zones were conducted to aid in identifying mechanisms and environmental pathways controlling PPCP loading and distribution at Great Lakes MWP sampling locations. The “Results and discussion” section of this study addresses the magnitude and environmental occurrence of PPCPs measured in dreissenid mussels, and further highlights the complex nature of these emerging contaminants, as we assess and discuss their detection basin-wide, and at various point source/non-point source, and site land-use gradients. This study further characterizes PPCP tissue body burden levels and provides insights into which compounds might potentially influence and pose adverse ecotoxicological effects and endpoints in lower trophic organisms. Study implications including follow-up and comparative studies are also discussed in an effort to prioritize contaminants to be monitored, thus providing the most efficient use of resources and funds that can support best management practices (BMPs), and measures in reducing and abating the continued occurrence of PPCPs in stressed aquatic environments.

### PPCP occurrence and concentration

**Fig. 3:**
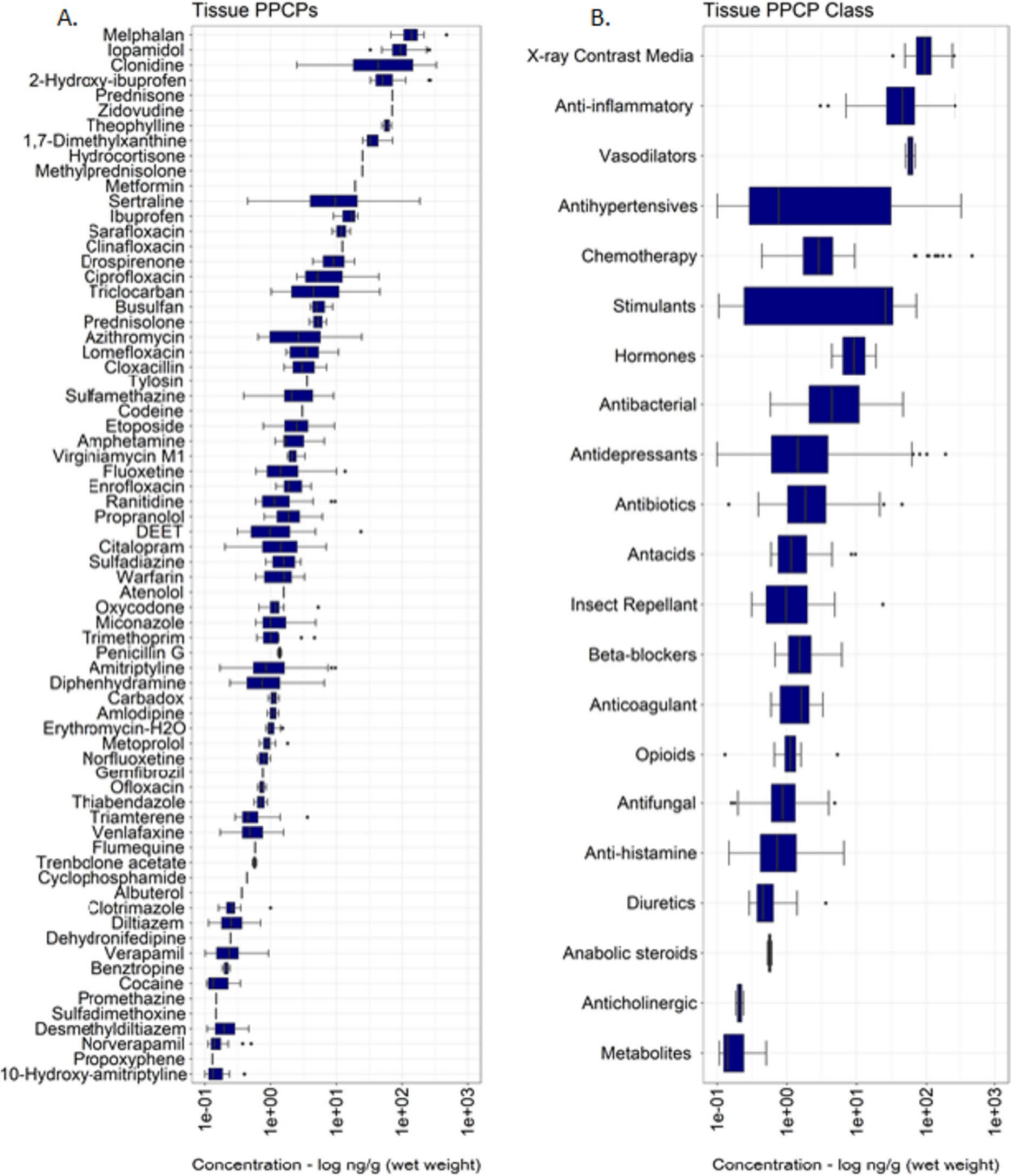
Box and whisker plots showing PPCP concentrations (log ng/g (wet weight)) detected in dreissenid mussels sampled between 2013 and 2018. Figure A depicts PPCP concentrations summarized by compounds in descending order based on highest to lowest mean concentration, while figure B groups the same compounds and depicts PPCP concentration summarized by compound class in descending order based on highest to lowest mean concentration. The *x* axis (log scaled) represents several orders of magnitude difference in PPCP compounds and compound class concentrations quantified in dreissenid mussel tissue samples. Additional information is provided in Table S4

The magnitude and detection frequency of individual PPCPs quantified in dreissenid mussels from sites in the Great Lakes is summarized in Table S4 (Supplementary Information). PPCPs were detected across all Great Lakes sites, mainly as complex mixtures, with 4 to 28 compounds detected at one or more mussel sampling location. Of the 141 compounds analyzed in this study, 70 (46.6%) human and veterinary PPCPs were detected at least once in dreissenid mussel tissue, with single-sample concentrations across all sites ranging from 0.057 to 475 ng/g (wet weight). Melphalan, a chemotherapy (alkylating cytostatic agent) drug used to treat cancer (James et al., 2020), was detected at the highest mean concentration (162.1 ng/g wet weight; Fig. 3A) in mussels across all sampling locations. Although infrequently detected in mussel tissue (< 10%), melphalan concentrations (range: 67.8–475 ng/g wet weight) measured in this study are suggested to be within estimated clinical doses administered to cancer patients (a dose of 200 mg/m^2^ is a standard preparative conditioning treatment dose, but a dose of 140 mg/m^2^ is often used in patients [e.g., older patients] perceived to be at risk of excess toxicity; Srour et al., 2019), thus making the detection of these compounds in mussel tissue biologically relevant (James et al., 2020). While melphalan has been reported in previous studies to be susceptible to photodegradation and is moderately biologically labile (Franquet-Griell et al., 2017; James et al., 2020), the detection of these cytostatic micro-pollutants in freshwater systems at high concentration is of particular interest, due to their potential ecotoxicological/adverse effects (e.g., cytotoxic, genotoxic, mutagenic, and teratogenic effects) in non-target aquatic organisms (Buschini et al., 2003; Jureczko & Kalka, 2020; Yadav et al., 2021).

**Fig. 4:**
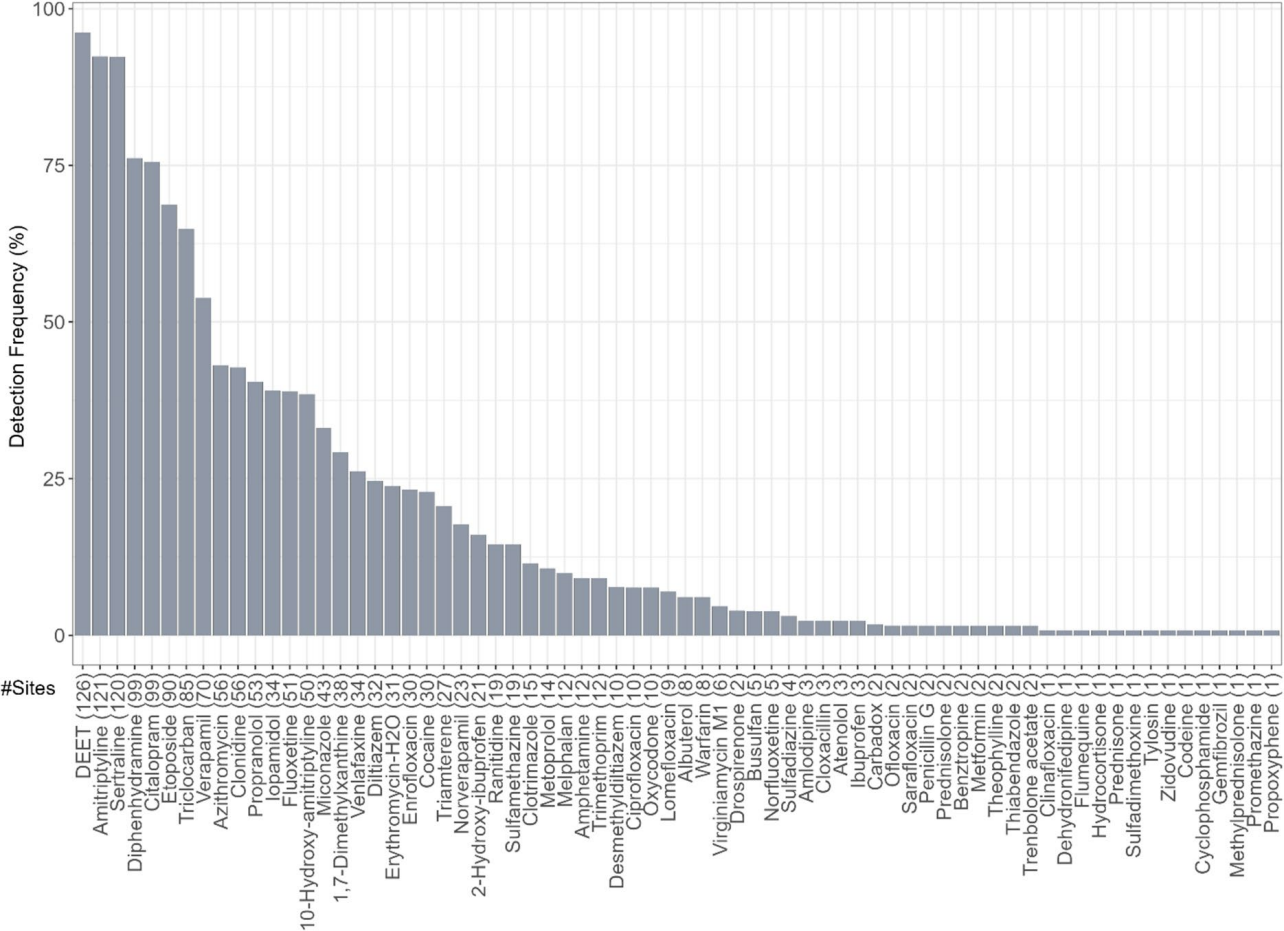
Detection frequency (%) for PPCP compounds detected at least once in dreissenid mussels sampled at Great Lakes study sites between 2013 and 2018. The total number of compounds detected per site is depicted in adjoining parentheses. Compounds are ordered by those most frequently to least detected

Iopamidol, a nonionic and monomeric iodinated contrast media (ICM) compound (Mestre et al., 2014), was also detected at relatively high mean concentration in mussel tissue, followed by clonidine, 2-hydroxy-ibuprofen, prednisone, zidovudine, theophylline and 1,7-dimethylxanthine, hydrocortisone, metformin, methylprednisolone, and sertraline (Fig. 3A; Table S4). As for other PPCPs assessed in this study, approximately 76% (53/70) were measured at low mean concentrations (< 10 ng/g wet weight), indicating either active dilution or low uptake occurring in dreissenid mussels at sampled locations. Overall, verapamil (antihypertensives), triclocarban (antibacterial), etoposide (chemotherapy), citalopram (antidepressants), diphenhydramine (anti-histamine), sertraline (antidepressants), amitriptyline (antidepressants), and DEET (*N,N*-diethyl-meta-toluamide; insect repellant) were among the most ubiquitous pharmaceuticals (frequency; > 50%) detected in dreissenid mussels (Fig. 4).

All PPCPs measured during the study period were prioritized and grouped into 27 primary therapeutic class in an effort to understand the complexity behind PPCP mixtures and chemical profile detected in mussels at MWP sites. As shown in Fig. 3B and Table S4, compositional differences were observed among PPCPs examined in this study, with 1 to 18 compounds detected within some classes of pharmaceuticals. Overall, antihypertensives (4 compounds), anti-inflammatories (7 compounds), antidepressants (7 compounds), and antibiotics (18 compounds) accounted for majority of the total PPCPs quantified in mussel tissue (Table S4). Several classes of pharmaceuticals, including chemotherapy, anti-histamine, antibacterial, antidepressants, and insect repellant, were among the compound groups most frequently (> 50%) detected in this study, thus signifying their ubiquity and widespread use within the Great Lakes Basin.

On average, measured PPCP concentrations varied for some classes of pharmaceuticals, spanning between 1 and > 2 orders of magnitude. Non-steroidal anti-inflammatory drugs (NSAIDs; Izadi et al., 2020), X-ray contrast media drugs, vasodilators, antihypertensives, and chemotherapy compounds were among the classes of pharmaceuticals observed at the highest mean concentrations in this study (Fig. 3B). The sub-lethal effects on non-target organisms due to long-term exposures from pharmaceuticals including NSAIDs, X-ray contrast media drugs, and chemotherapy compounds detected in this study cannot be neglected. Previous studies have shown NSAID exposure at low/environmentally relevant concentrations can cause a variety of adverse effects at molecular, biochemical, and cellular level (Contardo-Jara et al., 2011; Gómez-Oliván et al., 2014; Parolini, 2020). Elevated concentrations of X-ray contrast media drugs including iopamidol are often linked to their excretion from the human body in an unmetabolized form (greater than > 90% excreted may be unmetabolized within 72–96 h after administration; Matsushita et al., 2015) and an incomplete/low elimination during wastewater treatment process (removal efficiency < less than 35%), which is mainly attributed to the high biological stability and solubility of these compounds (Fabbri et al., 2016; Gao et al., 2019). Equally important, iopamidol detection in freshwater systems at elevated concentrations is also of particular interest due to their reaction with aqueous chlorine, resulting in the formation of highly cytotoxic and genotoxic iodinated disinfection by-products (I-DBPs; Ackerson et al., 2020; Postigo et al., 2018).

As a class, antidepressants (7 compounds; Table S4) showed significant variability, with individual compound concentrations varying by 1 to 2 orders of magnitude. For example, fluoxetine and sertraline, widely prescribed selective serotonin reuptake inhibitor (SSRI) drugs, were measured at the highest mean concentrations. Likewise, citalopram and amitriptyline were among those antidepressants frequently detected (detection frequency; > 92%) in mussels during the 2013–2018 sampling period. The frequent detection of these psychoactive drugs in this study might be explained by their extensive use in treating various depressive disorders (Yang et al., 2019). In a 2015–2018 survey, 13.2% of adults in the USA reported taking antidepressants, with fluoxetine and sertraline among the most commonly prescribed (Brody & Gu, 2020; Luo et al., 2020). Furthermore, the elevated concentration and frequent detection of antidepressants in this study are indicative of their incomplete breakdown in conventional wastewater systems and release from fugitive domestic wastewater sources (Metcalfe et al., 2010; Thompson & Vijayan, 2022).

Antibiotics are among the most commonly prescribed pharmaceuticals worldwide (Van Boeckel et al., 2014) and are mainly prescribed for its mixed-use in human and veterinary medicine, in controlling/treating bacterial infections among other uses (Kovalakova et al., 2020). Among the antibiotics examined in this study, the mixed-use antibiotic azithromycin (a macrolide antibiotic used in the treatment of bacterial infections; Oliver & Hinks, 2020) and ciprofloxacin (a fluoroquinolone antibiotic widely prescribed and mainly used to control urinary tract and respiratory infections; Hashemzadeh et al., 2021) were detected at the highest concentrations in mussel tissue samples (24.4 and 44.5 ng/g wet weight; Table S4). However, of the 18 human and veterinary antibiotics detected in mussel tissue, approximately 83% (15/18) were seldom detected (detection frequency < 20%; Table S4) in this study, which likely result from the low uptake of these pollutants in mussel tissue (Bris et al., 2004). Antibiotic residue detection in aquatic environment has been shown to be a major concern due to the development of antibiotic resistance genes (ARGs) and antibiotic-resistant bacteria (ARB). Previous studies have shown antibiotic resistance genes pose elevated risk due to antibiotic resistance and reduce therapeutic efficacy against various human and animal pathogens (Ebele et al., 2017; Felis et al., 2020; Kumar et al., 2019). Certainly, the detection of micro-pollutants including antidepressants and antibiotics in large lentic (Ferguson et al., 2013) and lotic (e.g., riverine; Larson et al., 2013b) systems such as the Great Lakes basin warrants further examination of the potential effects of these contaminants.

### PPCP spatial patterns

**Fig. 5:**
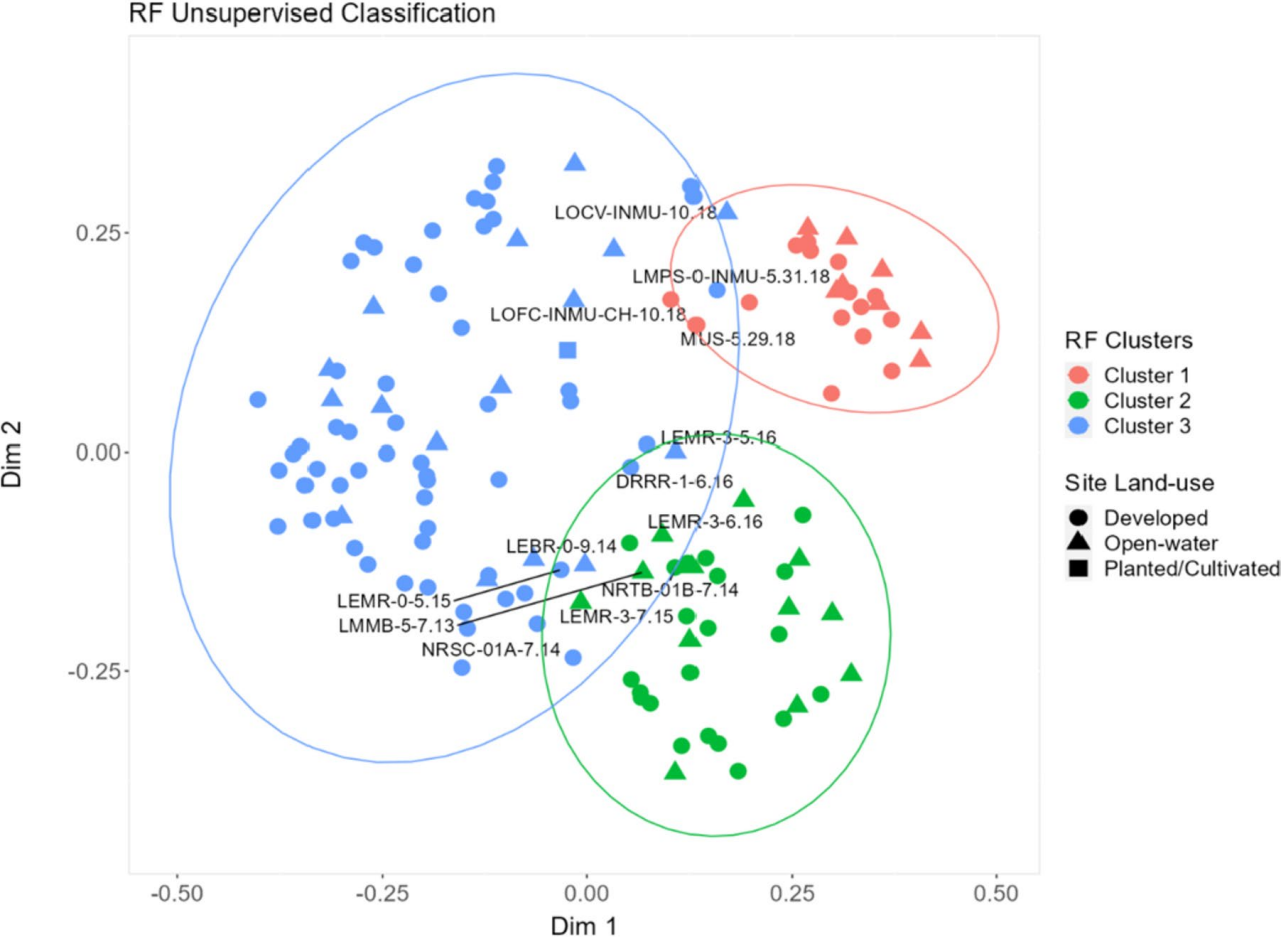
Spatial grouping and clusters for Great Lakes MW PPCP sampling locations resulting from random forest (RF) unsupervised classification and cluster analyses. Ellipses represents the 95% confidence intervals for each RF cluster. RF classification results represent three major clusters (clusters 1–3) based on PPCP presence/absence (1/0) and composition profile across mussel sampling locations. Planted/cultivated land cover category was dominant (> 30%) at one study site (NRYT-INMU-CH-10.18; see Fig. 2). Additional information on study sites that overlap clusters and fall within the 95% confidence interval is provided in Table S10

**Fig. 6:**
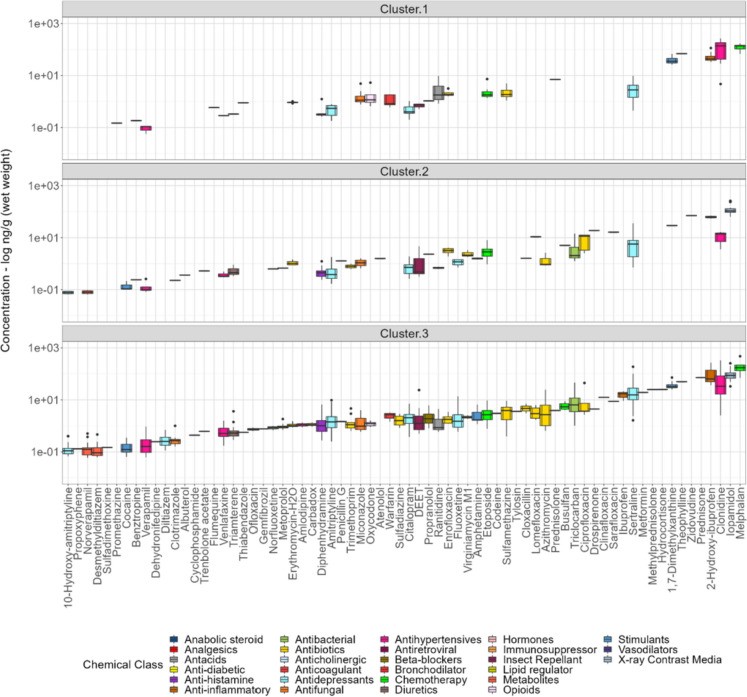
Box and whisker plots showing PPCP concentration (log ng/g (wet weight)) profile for compounds observed in individual clusters derived from unsupervised random forest (RF) classification. PPCP compounds are ordered by lowest to highest concentration in each RF cluster. Cluster 3 represents the group with the most predominant PPCP mixtures and contained the greatest chemical load and composition observed between clusters. Additional information is provided in Table S5

Overall differences in detected PPCP composition and concentration were assessed and compared to find patterns in mussel tissue data using a combination of RF unsupervised classification, followed by cluster analyses. Results from the cluster analysis revealed three different clusters (Fig. 5 and Fig. S1). On average, PPCP concentration varied between clusters, with relatively higher mean concentrations observed for PPCPs examined in cluster 1 (mean 16.6 ng/g ww; range 0.11–266 ng/g wet weight), compared to concentrations measured in cluster 2 (mean 8.16 ng/g ww; range 0.086–261 ng/g wet weight) and cluster 3 (mean 12.1 ng/g ww; range 0.13–475 ng/g wet weight; Fig. 6; Table 1 and Fig. S1; Table S5), demonstrating significant differences in PPCP contaminant levels measured in mussels across the Great Lakes Basin. However, differences in cluster 1–3 concentrations were not statistically significant (Kruskal–Wallis; *p* > 0.05). Furthermore, 17 compounds were consistently detected co-occurring across all three clusters, with overall PPCP composition and loading higher for sites sampled in cluster 3 (91%; 64/70), compared to cluster 1 (60%; 42/70) and cluster 2 (39%; 27/70), respectively (Table S5). Similarly, cluster 3 represented the group with the highest total PPCP concentration (8860.5 ng/g wet weight), compared to cluster 1 (6660.4 ng/g wet weight) and cluster 2 (3133.8 ng/g wet weight), respectively (Table S7).

**Table 1:** Summary of dreissenid mussel tissue PPCP concentrations (ng/g (wet weight)) detected in RF clusters (1–3). Additional information is provided in Fig. 6 and Table S3 (Supplementary Information)

Category	Count	Stdev	Minimum	Median	Mean	Maximum
			ng/g ww	ng/g ww	ng/g ww	ng/g ww
Cluster 1	189	33.9	0.066	1.20	16.6	269
Cluster 2	249	27.8	0.178	1.63	8.16	265
Cluster 3	1101	37.1	0.057	1.52	12.1	475

Additional analysis revealed differences in mussels sampled at inshore (river-tributary-harbor complexes) and offshore sampling locations. On average, overall PPCP concentrations varied across inshore and offshore sampling locations, with the highest mean concentrations observed in mussels from tributary sites sampled in cluster 1 (58.6 ng/g wet weight; Table 2). However, differences in inshore and offshore sampling locations PPCP concentrations were not statistically significant (Kruskal–Wallis; *p* > 0.05). Overall mean concentrations measured in mussels from harbor (30.1 ng/g wet weight) and offshore sites (32 ng/g wet weight) in cluster 1 were 1 to 2 orders of magnitude higher than mussels from cluster 2 and cluster 3, indicating active/fresh PPCP inputs (primary source), as well as pseudo-persistence occurring at these sampling locations. Likewise, additional comparison revealed elevated mean concentration in mussels from river sites (17.2 ng/g wet weight) in cluster 2, and mussels from river, harbor, and offshore sites sampled in cluster 3 (range: 9.84–10.3 ng/g wet weight; Table 2 and Fig. S2). Overall, measured concentration for 9 PPCPs (1,7-dimethylxanthine, 2-hydroxy-ibuprofen, theophylline, ciprofloxacin, clonidine, iopamidol, sertraline, triclocarban, and melphalan) was higher in mussels across all examined inshore and offshore sampling locations (Table S5). However, with the exception of iopamidol, the magnitude for 7 compounds (melphalan, 1,7-dimethylxanthine, 2-hydroxy-ibuprofen, ciprofloxacin, clonidine, sertraline, and triclocarban) was higher in mussels from inshore (i.e., harbor and river sites) and offshore sites in cluster 3, compared to cluster 2 and cluster 1.

**Table 2:** Summary of the PPCP concentrations (ng/g (wet weight)) detected in dreissenid mussel tissue sampled at inshore (harbor, river, tributaries) and offshore sampling locations between 2013 and 2018

Category	Sampling locations	Count	Stdev	Min	Median	Mean	Max
				ng/g ww	ng/g ww	ng/g ww	ng/g ww
Cluster 1	Harbor	98	59.1	0.057	1.78	30.1	266
	River	27	25.1	0.109	1.89	11.2	123
	Tributary	4	58.6	1.58	24.8	58.6	183
	Offshore	60	55.2	0.182	1.97	32	265
Cluster 2	Harbor	74	13.5	0.072	1.42	5.3	92.7
	River	84	41.6	0.066	1.69	17.2	261
	Tributary	11	4.59	0.101	2.94	4.61	13.1
	Offshore	80	26.4	0.066	1.02	5.16	236
Cluster 3	Harbor	353	29.1	0.07	1.8	9.84	332
	River	488	30.7	0.061	1.32	10.3	269
	Tributary	59	8.34	0.07	1.01	3.82	46.2
	Offshore	201	39.8	0.059	1.4	10.3	475

With the exception of cluster 2, measured concentrations for 2-hydroxy-ibuprofen (range: 32.9–269 ng/g wet weight), clonidine (range: 3.6–332 ng/g wet weight), and melphalan (range: 67.8–475 ng/g wet weight) were highest across cluster 1 and cluster 3, spanning several orders of magnitude when compared to other PPCP compounds assessed in this study (Table S5). Additional examination revealed 2-hydroxy-ibuprofen (anti-inflammatory), clonidine (antihypertensives), and melphalan (chemotherapy) elevated concentrations across cluster 1 and cluster 3 are indicative of their incomplete breakdown and release from wastewater treatment processes and medical discharges, since these pharmaceuticals are used extensively to treat cancer, hypertension (e.g., regulate blood pressure), and other related ailments (Ebele et al., 2017; Godoy et al., 2015; Yadav et al., 2021). Overall, this study confirms PPCPs detected in mussels from inshore and offshore sites are similar in complexity and occurrence to other comparative studies that have examined PPCPs throughout the Great Lakes (Baker et al., 2022; Banda et al., 2020b; Choy et al., 2017; Custer et al., 2020). Equally important, this study further provides evidence of emerging contaminant concentrations and composition strong variations that can occur between inshore and offshore (i.e., open/deep lake) sampling locations.

### PPCP basin-wide and reference site assessment

Basin-wide, we detected significant differences for the majority of PPCP compounds measured in this study, although more sites were sampled in Lakes Michigan (67 sites) and Erie (29 sites), compared to other lakes (Lake Huron and Lake Ontario) and connecting channels (inclusive of all sites in the Detroit River and Niagara River), due in part to PPCP sampling objectives. For example, PPCP concentration and composition varied among lakes, connecting channels, and reference sites, with relatively higher mean concentration detected in mussels from Lake Huron (39.8 ng/g wet weight), compared to Lakes Erie (11.7 ng/g wet weight), Michigan (14.0 ng/g wet weight), Ontario (21.0 ng/g wet weight), the Niagara (5.10 ng/g wet weight), and Detroit River (5.95 ng/g wet weight) connecting channels, respectively (Table 3 and Fig. S3). Overall, PPCPs were mainly detected as complex mixtures basin-wide, with 4 (reference site; NRNF-1–7.14) to 28 (LMMB-01-INMU-6.18) compounds detected in mussels at one or more sampling location. These results indicate that PPCP chemical composition generally differed among MWP sites basin-wide, with some sites experiencing elevated contaminant exposure during the 2013–2018 study period. In addition to this study, several comparative studies have shown PPCPs are also occurring as complex mixtures in surface water (Baker et al., 2022; Baldwin et al., 2016; Blair et al., 2013; Ferguson et al., 2013; Pronschinske et al., 2022) and tissue samples (i.e., mussels, fish, and tree swallows [*Tachycineta bicolor*]; Banda et al., 2020; Cipoletti et al., 2019; Custer et al., 2020; Deere et al., 2020; de Solla et al., 2016; Woolnough et al., 2020) in the Great Lakes region. As shown in a previous study, the detection of these compounds as complex mixtures in the Great Lakes is of concern, due to their constituents, which were formulated to act on specific molecular targets in humans (Maloney et al., 2022).

**Table 3:** Summary of the PPCP concentrations (ng/g (wet weight)) detected in dreissenid mussel tissue sampled at Lake Michigan, Lake Huron, Lake Erie, Lake Ontario, Detroit, and Niagara River connecting channels (*), and designated MW reference sites between 2013 and 2018. Additional information is provided in Table S5

Category	Count	Stdev	Minimum	Median	Mean	Maximum
			ng/g ww	ng/g ww	ng/g ww	ng/g ww
Detroit River (*)	82	9.55	0.062	1.31	5.95	41.2
Lake Erie	251	31.7	0.061	1.49	11.7	261
Lake Huron	20	57.3	0.182	2.53	39.8	183
Lake Michigan	762	37.2	0.057	1.73	14.0	332
Lake Ontario	65	69.7	0.065	1.35	21.0	475
Niagara River (*)	191	21.9	0.059	1.03	5.10	269
Reference sites	168	26.3	0.067	1.22	7.62	236

A lake-lake comparison revealed antihypertensives, antidepressants, anti-histamine (diphenhydramine), insect repellant (DEET), chemotherapy (Etoposide), and antibacterial (triclocarban) were among the classes of pharmaceuticals most frequently detected in mussel’s basin-wide. PPCPs frequently detected in mussels represent a wide variety of therapeutic uses, with most previously reported in comparative surface water (Baker et al., 2022; Blair et al., 2013; Ferguson et al., 2013; Pronschinske et al., 2022) and tissue studies (Banda et al., 2020; Custer et al., 2020; Deere et al., 2020; de Solla et al., 2016; Woolnough et al., 2020) in the Great Lakes region. Overall, cumulative tissue concentrations (sum of detected PPCPs) ranged by several orders of magnitude (range: 3.6–674.9 ng/g ng/g wet weight) per site, with the highest total concentrations (> 350 ng/g wet weight) measured in mussels from sites sampled in Lake Michigan (MUS-6.26.18 and LMMB-01-INMU-6.18; Fig. S5 and Table S7). This finding is consistent with results from previous studies that reported elevated PPCP levels in fish (Banda et al., 2020) and surface water (Elliott et al., 2017; Ferguson et al., 2013; Pronschinske et al., 2022) were highest in Lake Michigan.

A broader assessment of PPCPs detected in mussels from designated offshore reference sites revealed significant variation in PPCP frequency, composition, and concentration. Of the 70 PPCPs quantified in this study, 37 (53%) compounds were detected in mussels from offshore reference sites (Fig. S4 and Table S6). Etoposide (68%), diphenhydramine (79%), citalopram (79%), DEET (89%), amitriptyline (89%), and sertraline (100%) were among the PPCPs most frequently detected in mussels from designated reference sites. Overall PPCP concentrations ranged from 0.067 to 236 ng/g (wet weight), with the highest total concentrations measured in mussels from reference sites in the Milwaukee Estuary (LMMB-5-S5-8.17) and western Lake Erie basin (Maumee Lighthouse: LEMR-3–6.15; Fig. S5 and Table S7). On average, approximately 78% (29/37) of the PPCPs measured in mussels from designated reference sites were measured at relatively low concentrations (< 5 ng/g wet weight). The detection of PPCPs at environmentally low concentrations, and also as complex mixtures in non-target lower trophic organisms including dreissenid mussels, especially in offshore zones of large water bodies such as the Great Lakes remains poorly understood (Blair et al., 2013). Thus, the toxicological effects and endpoints resulting from PPCPs long-term (i.e., chronic exposure), and low-level exposures in dreissenid mussels and other aquatic biota, may warrant additional consideration and future assessments of these emerging contaminants in the Great Lakes.

In general, several compounds including 1,7-dimethylxanthine, sertraline, metformin, clonidine, and iopamidol were measured at the highest mean concentration in mussels across all designated reference sites (> 10 ng/g wet weight; Fig. S4 and Table S6). Interestingly, clonidine, 1,7-dimethylxanthine, and iopamidol concentrations measured in mussels from offshore reference sites were similar or equal to chemical signatures observed at some inshore sites. Elevated concentrations detected at offshore reference sites, which are well beyond major pollution sources (e.g., Maumee Lighthouse-3, ~ 10 km away from nearest WWTP and storm-water outfall), are indicative of some PPCPs pseudo-persistence, recalcitrance to degradation (e.g., abiotic and biotic transformations), and active offshore contaminant transport in the Great Lakes Basin (Blair et al., 2013; Helm et al., 2012; Kimbrough et al., 2018; Patel et al., 2019). This further highlights the importance of bio-monitoring programs such as the MWP, and studies such as this in sampling emerging contaminants on a larger spatial scale, even at offshore lake complexes to better understand the magnitude and distribution of these organic contaminants in large freshwater systems.

### PPCP discharge type assessment

**Fig. 7:**
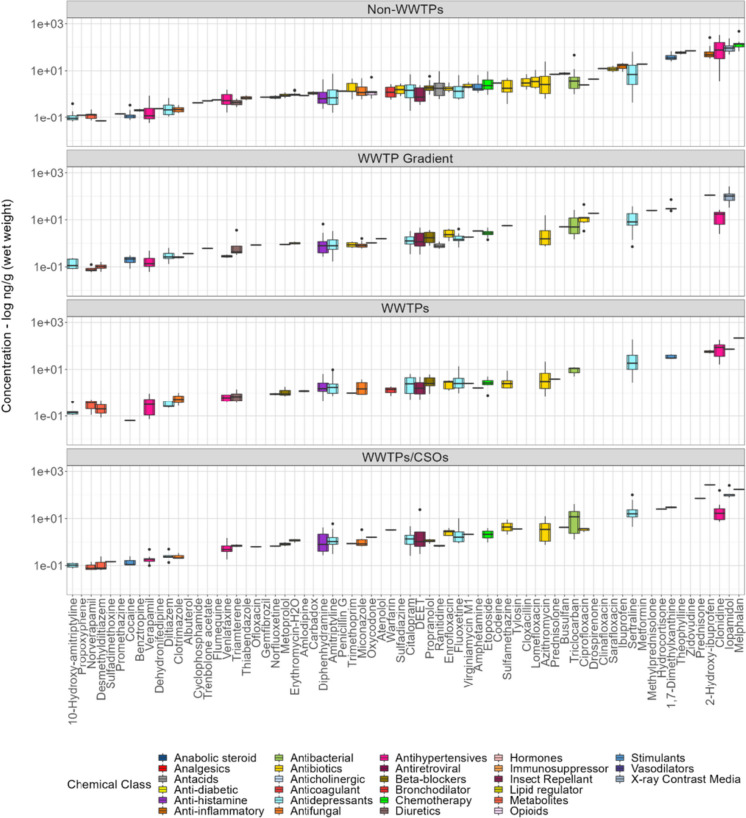
Box and whisker plots depicting detected concentrations (log ng/g (wet weight)) for individual PPCPs detected in dreissenid mussels sampled at sites proximate to point source discharge (WWTPs and WWTPs/CSOs), sites downstream and along gradients of wastewater discharge (WWTP Gradient), and non-WWTP sites (sites not influenced by WWTPs or CSOs) during 2013–2018. PPCP compounds are ordered by lowest to highest concentration in each discharge category. Additional information is provided in Table S8

The concentration profile for PPCPs measured in dreissenid mussels at major discharge types, including sites proximate to WWTPs only, sites proximate to both WWTP and CSO point source discharge, sites downstream and along gradients of wastewater discharge, and sites not influenced by either WWTP or CSO discharge (i.e., non-WWTPs), is presented in Fig. 7 and Table S8. Overall, PPCP concentrations varied among the major discharge types assessed in this study, with relatively higher mean concentration observed in mussels from non-WWTP sites (13.4 ng/g wet weight; Table 4). However, differences in discharge type mean concentrations were not statistically significant (Kruskal–Wallis; *p* > 0.05). Compared to the other major discharge types, PPCP loading and composition were also higher in mussels from non-WWTP sites (62/70; Table S8), thus demonstrating non-point/diffuse sources might also be important pollution pathways and routes of exposure for PPCPs detected within the Great Lakes Basin. Equally important, the highest total PPCP concentrations (summed by their respective compound class) were measured at non-WWTP sites (11,899.1 ng/g wet weight), compared to sites sampled proximate to WWTPs (1883.4 ng/g wet weight), sites downstream and along wastewater gradients (2149.1 ng/g wet weight), and sites sampled proximate to WWTPs/CSOs (2547.0 ng/g wet weight), respectively.

**Table 4:** Summary of dreissenid mussel PPCP concentration (ng/g (wet weight)) measured at major discharge types including sites sampled proximate to point source discharge (i.e., WWTPs and CSOs), sites downstream and along gradients of wastewater discharge, and sites without wastewater influence (non-WWTPs). Additional information is provided in Table S8

Category	Count	Stdev	Minimum	Median	Mean	Maximum
			ng/g ww	ng/g ww	ng/g ww	ng/g ww
Non-WWTPs	891	39.1	0.057	1.55	13.4	475
WWTP gradient	278	23.4	0.059	1.28	7.73	261
WWTPs	149	32.8	0.064	1.89	12.6	217
WWTPs/CSOs	221	33.5	0.065	1.36	11.5	269

While PPCPs relative concentration and composition are expected to be higher at sites in proximity to point sources (Apeti et al., 2018), comparative studies have shown fugitive release from leaking septic systems, landfills/landfill leachate, urban runoff, agricultural runoff following the land-applied of manure and/or biosolids, runoff from CAFO facilities, and overland flow of contaminants can contribute to elevated PPCP composition and concentration detected at non-WWTP zones (Adams et al., 2014; Baldwin et al., 2016; Elliott et al., 2017; Fairbairn et al., 2016; Ferrey et al., 2015; Pronschinske et al., 2022). In a follow-up study, Deere et al. (2021) demonstrated that a large percentage of high priority pharmaceuticals was detected at remote regions (e.g., non-WWTP zones) due to factors such as atmospheric wet deposition and the persistence of these organic pollutants. On average, the highest PPCP loading and concentration were measured in mussels from non-WTTP sites in Lake Michigan (MUS-6.26.18 and LMMB-01-INMU-6.18). As shown in prior studies conducted by Blair et al. (2013) and Li et al. (2017), elevated PPCP composition and concentration detected in Milwaukee Estuary, Lake Michigan, are not uncommon, as urban runoff and river discharge from the Menomonee River, Kinnickinnic River, and Milwaukee River confluence is viewed as potential sources of elevated PPCP composition and concentration.

Overall, a total of 29 PPCPs were found recurring in mussels across all site discharge types, which further highlight the ubiquity and persistence of these contaminants across all sampling types and locations in the Great Lakes Basin. In addition, 9 PPCPs (atenolol, benztropine, cyclophosphamide, flumequine, penicillin G, prednisolone, promethazine, thiabendazole, and zidovudine) were only detected in mussels from non-WWTP sites, which were not detected at any other discharge type assessed in this study (Table S8). In general, 5 PPCPs (1,7-dimethylxanthine, 2-hydroxy-ibuprofen, iopamidol, clonidine, and melphalan) were consistently measured at relatively high mean concentrations (> 30 ng/g wet weight; Fig. 7) in mussels across all site discharge types. Similar to studies conducted by de Solla et al. (2016) and Deere et al. (2020), this study detected iopamidol (range: 77.7–254 ng/g [wet weight]), melphalan (range: 172–217 ng/g [wet weight]), and 2-hydroxy-ibuprofen (range: 47.9–269 ng/g [wet weight]) concentrations relatively higher at sites sampled in proximity to WWTP/CSO point discharge.

Based on a prior study of 1448 municipal WWTPs found within the US and Canadian portion of the Great Lakes Basin coastal zone that discharge treated effluent to the Great Lakes Basin International Joint Commission (2009), most of the secondary and conventionally designed WWTPs are capable of achieving reductions for some emerging contaminants. Thus, the above finding suggests the type of wastewater treatment system, incomplete elimination during conventional wastewater treatment processes, low in-stream dilution, and hydrodynamic flushing and residence time are important drivers for various pharmaceuticals including 2-hydroxy-ibuprofen, melphalan, and iopamidol detected at mussel sampling locations (Baker et al., 2022; Castellano‐Hinojosa et al., 2023; Deere et al., 2021; de Solla et al., 2016; Ribbers et al., 2019). Additional correlation analysis revealed strong positive association between several classes of pharmaceuticals and point source parameters (i.e., WWTPs, WWTP effluent flow, and CSOs). Specifically, strong correlations were observed between 11 classes of pharmaceuticals (antacids, anti-histamines, antihypertensives, chemotherapy, diuretics, insect repellant, metabolites and transformation products (TPs), opioids, stimulants, X-ray contrast media, and antibacterial) and site point source/wastewater parameters (Spearman’s *rho* (*ρ*) = 0.5007–0.9341; Table S12).

Similar to results presented in Fairbairn et al. (2016) and Deere et al. (2020), four mixed-use pharmaceuticals (hydrocortisone, prednisone, sulfadimethoxine, and tylosin) were only detected in mussels from WWTP/CSO sites in this study (Table S8). The presence of macrolide and sulfonamide antibiotic residue, such as tylosin and sulfadimethoxine, in freshwater environments is more related to agricultural/non-urban activities, which includes extensive use in cattle, swine, and beef confined animal feeding operations (CAFO; De Liguoro et al., 2007; Fairbairn et al., 2016; Kaczala and Blum, 2016). However, tylosin (brand name, Tylan) and sulfadimethoxine (brand name, Albon) detection in mussels from WWTP/CSO discharge sites in the lower Maumee (LEMR-1–6.16) and the Detroit riverine system (River Rouge-2–6.16) is indicative of their mixed-use in both non-urban and urban/residential settings (Grześkowiak et al., 2015; Papich., 2020).

Interestingly, among the classes of pharmaceuticals examined in this study, antifungal, anti-histamine, beta-blockers, diuretics, metabolites, and opioids displayed similar chemical profile across all major discharge types (Fig. S6 and Table S9), which is likely indicative of the environmental fate and attenuation dynamics (e.g., abiotic transformation, photodegradation, and biodegradation processes) occurring across these discharge types. Similar to our findings at wastewater impacted sites (Custer et al., 2020; de Solla et al., 2016; Elliott et al., 2018), sites downstream and along wastewater gradients (Cipoletti et al., 2020; Jorgenson et al., 2018; Thomas et al., 2017), and non-WWTP sites (Deere et al., 2020), the detection of these contaminants across major discharge types in this study highlights the acute and sublethal risk posed by these classes of emerging contaminants on non-targeted organisms in the Great Lakes Basin.

Comparable to studies conducted in the Great Lakes (Baker et al., 2022; de Solla et al., 2016; Muir et al., 2017; Woolnough et al., 2020), across North America (Bradley et al., 2017; James et al., 2020), and Europe (Álvarez-Muñoz et al., 2015), this study detected similar patterns in PPCP concentrations for antibacterial (i.e., triclocarban wastewater indicator compound), stimulant (i.e., 1,7-dimethylxanthine, amphetamine, and cocaine), and chemotherapy pharmaceuticals at sites in proximity to WWTP and CSO discharges (Fig. S6 and Table S9). Thus, input from WWTP and CSO discharge is considered an important anthropogenic driver for these organic pollutants elevated concentration measured in mussels across these major discharge types. In addition, Spearman’s correlation results further revealed antibacterial, as well as anti-histamine, beta-blockers, diuretics, and PPCP transformation products (TP), were strongly correlated to site population estimates (Spearman’s *rho* (*ρ*) = 0.707–0.799; Table S12), suggesting local usage, pharmaceutical consumption patterns (e.g., seasonal usage patterns; Boogaerts et al., 2021), and wastewater treatment loading capacity are drivers for to the widespread detection of these contaminants (Khasawneh and Palaniandy., 2021). Although PPCP chemical interactions across major discharge types were not addressed in the current study, the sublethal effects for most contaminants detected in mussels across the major discharge types in this study are unknown. As such, follow-up investigations should be conducted to address potential environmental exposure and effects on non-targeted and lower trophic aquatic organisms.

### PPCP relationship to land-use gradients

Detailed information on mussel sampling locations land-use/land cover estimates are presented in Table S10. Additional information on PPCP magnitude and environmental occurrence at predominant developed and open-water sites are presented in Table S11. The overall pattern in PPCP distribution remained heterogeneous among land-use categories assessed in this study (Fig. S7 and Table S10). In addition, developed and open-water were among the dominant site land-use categories, as well as land-use groups depicting the highest correlation among the classes of pharmaceuticals examined in this study (Table S12). Planted/cultivated (agricultural) land cover category was dominant (> 32%) at one study site (NRYT-INMU-CH-10.18; Fig. 2 and Table S10). This site was not included in the final assessment of PPCPs detected at mussel study sites, since there were not enough planted/cultivated sites available for comparison and subsequent discussion in this study.

**Fig. 8:**
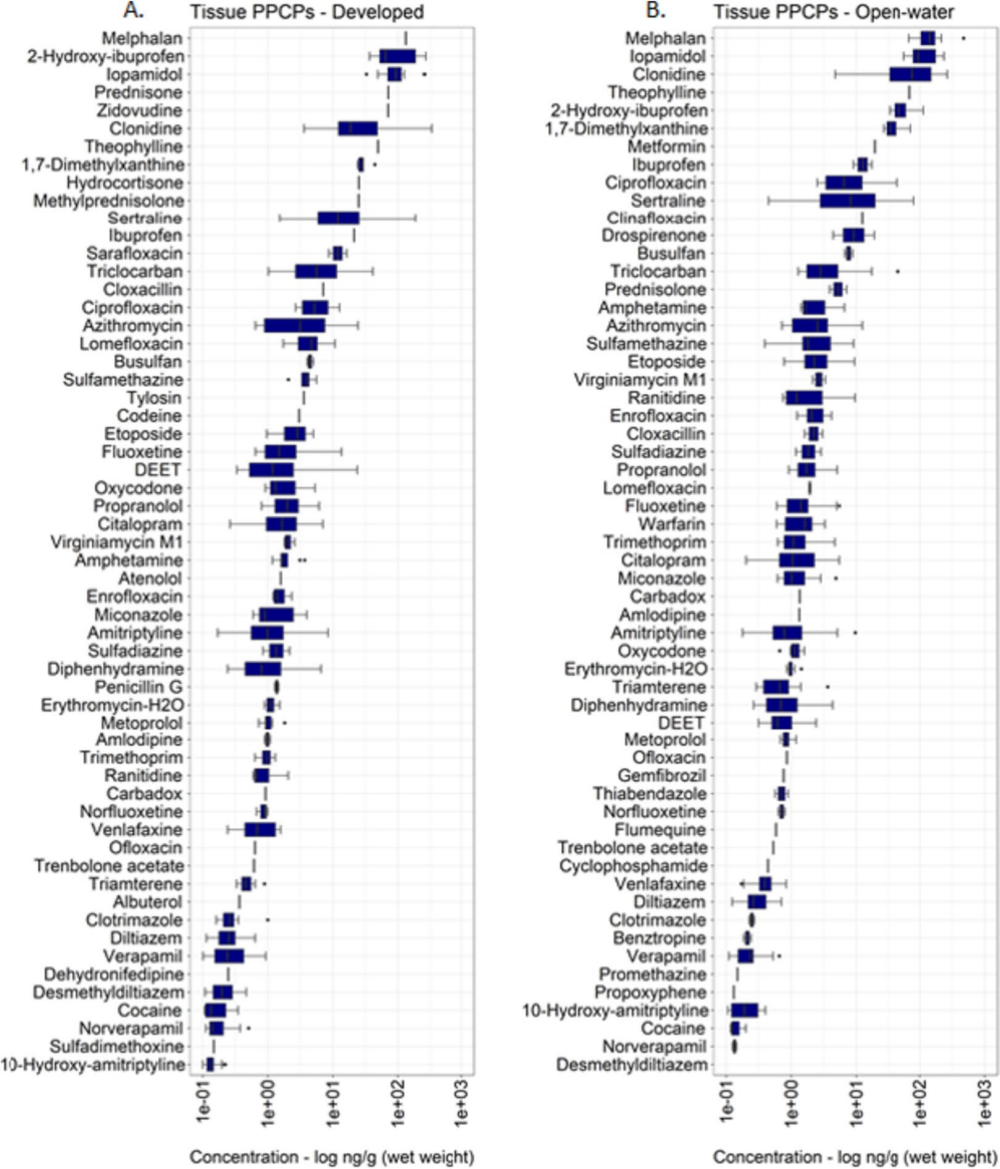
Box and whisker plots showing PPCP concentration (log ng/g (wet weight)) measured in dreissenid mussels from designated **A** developed (combined developed medium intensity; developed high intensity; developed, open space; developed, low intensity categories) and **B** open-water sites. PPCP concentrations summarized by compounds in descending order based on highest to lowest mean concentration. The *x* axis (log scaled) represents several orders of magnitude difference between PPCP concentrations quantified in dreissenid mussel tissue sampled at developed and open-water sites. Additional information is provided in Table S11

The magnitude for PPCPs examined at predominant developed (range: 0.147–332 ng/g wet weight) and open-water sites (range: 0.098–475 ng/g wet weight) sites varied, with several mixed-use and human-specific pharmaceuticals including azithromycin, etoposide, diphenhydramine, clonidine, verapamil, triclocarban, DEET, fluoxetine, citalopram, sertraline, and amitriptyline, among the compounds most frequently detected (> 50%) in mussels across developed and open-water sampling locations. However, PPCPs were detected at higher frequency in mussels from developed sites, compared to open-water sites. Relatively higher mean concentrations were observed in mussels from open-water sites (14.2 ng/g wet weight), compared to developed sites (9.72 ng/g wet weight; Fig. S8 and Table 5). However, differences in developed and open-water sites mean concentration were not statistically significant (Kruskal–Wallis; *p* > 0.05). Eight pharmaceuticals (triclocarban, theophylline, 1,7-dimethylxanthine, sertraline, iopamidol, 2-hydroxy-ibuprofen, clonidine, and melphalan) were among the compounds measured at relatively higher concentrations (> 40 ng/g wet weight; Fig. 8; Table S11) in mussels across developed and open-water sites. Interestingly, the magnitude for several compounds (clonidine, azithromycin, verapamil, etoposide, citalopram, triclocarban, sertraline, diphenhydramine, amitriptyline, and DEET) frequently detected (> 50%) in mussels from developed and open-water sites were similar or higher, compared to results and findings for mussels, fish, and bird tissue in other reported studies (Cipoletti et al., 2020; Custer et al., 2020; Deere et al., 2020; James et al., 2020; Meador et al., 2017; Muir et al., 2017).

**Table 5:** Summary of the PPCP concentrations (ng/g (wet weight)) detected in dreissenid mussel tissue sampled at predominant developed and open-water sites between 2013 and 2018. Additional information is provided in Table S9

Category	Count	Stdev	Minimum	Median	Mean	Maximum
			ng/g ww	ng/g ww	ng/g ww	ng/g ww
Developed	753	30.4	0.059	1.43	9.72	332
Open-water	775	39.6	0.057	1.58	14.2	475

The highest total PPCP concentrations (> 400 ng/g wet weight) were detected in mussels from open-water and developed sites in Lakes Ontario and Michigan (Fig. S5 and Table S7). Large urban clusters and higher residential/population density is likely related to the high variability and elevated PPCP concentrations detected in mussels from developed sites sampled in this study (Ferguson et al., 2013). As discussed in a previous study (Bai et al., 2018), developed/urban land cover types such as golf courses, dog parks, and recreation parks are likely major sources and environmental pathways for pharmaceuticals, due to increased surface runoff which introduces contaminants to urban surface waters, resulting in higher PPCP concentrations. Likewise, increased urban population density/residents often result in higher pharmaceutical consumption and usage, thus resulting in increased surface runoff and overland flow of contaminants to surface water (Wu et al., 2009). Similar to MacLeod and Wong (2010), this study detected positive relationship between higher PPCP concentrations and site population estimates (Table S7). Moreover, strong positive correlations were observed between several classes of pharmaceutical concentrations (chemotherapy, anticoagulant, x-ray contrast media, stimulants, insect repellant, beta-blockers diuretics, metabolites, anti-histamine, opioids, antacids, antihypertensives, and antibacterial) and developed and open-water land-use categories (Spearman’s *rho* (*ρ*) = 0.6386–0.913; Table S12), suggesting surrounding developed/urban and open-water land-use gradients are likely sinks and hotspots for these contaminants (Baldwin et al., 2016; Deere et al., 2021; Fairbairn et al., 2016).

Compared to other studies (Cipoletti et al., 2019; Custer et al., 2020), similar spatial patterns and chemical signatures were observed for some classes of pharmaceuticals examined at developed and open-water sites (Fig. S9 and Table S11). For example, antihistamine, antifungal, beta-blockers, diuretics, stimulants, metabolites/transformation products (TPs), and antidepressants were detected at similar or higher mean concentrations in mussels examined across developed and open-water sites, highlighting the complexity of PPCP source and anthropogenic pressures within the Great Lakes. Of interest, chemotherapeutic pharmaceuticals were detected at significantly higher mean concentrations in mussels at open-water sites, compared to developed sites. However, no statistically significant difference (Kruskal–Wallis *p* > 0.05) was observed between open-water and developed sites for melphalan concentrations. The highest concentrations of melphalan were found at developed and open-water sites in Lake Michigan/Black River (LMBR-0–5.18) and Lake Ontario/Cape Vincent (LOCV-INMU-10.18), respectively. Chemotherapeutic pharmaceuticals including melphalan body burden in tissue samples (i.e., fish and Bay mussels [*Mytilus trossulus*]) are documented for developed and open-water sites in the Great Lakes (Banda et al., 2020; Deere et al., 2020) and the Puget Sound (WA, USA; James et al., 2020). However, reported levels in the above studies are well below chemotherapeutic pharmaceuticals bivalve body burden values measured in this study (475 ng/g wet weight).

Similar patterns were observed for antihypertensive (range: 0.061–332 ng/g wet weight), and X-ray contrast media compounds (range: 49.2–261 ng/g wet weight), with mussels sampled at developed sites representing the highest concentrations detected in this study (Table S11). Compared to other developed sites examined in this study, X-ray contrast media concentrations were not statistically different across developed sampling locations (Kruskal–Wallis *p* > 0.05). However, higher concentrations were detected in mussels from sites sampled in the Maumee (Toledo WWTP/LEMR-1–6.15) and Ottawa (LEOT-2–6.15) riverine systems. Overall, X-ray contrast media magnitude detected in this study agrees with results reported in other studies conducted in the western Lake Erie basin (Cipoletti et al., 2020; Custer et al., 2020), northeastern Minnesota (Deere et al., 2020), and the Grand River watershed in southern Ontario (de Solla et al., 2016). Similarly, clonidine concentrations were not statistically different across developed sampling locations (Kruskal–Wallis *p* > 0.05). However, the highest average concentrations were observed in mussels from developed sites in Lake Huron, Thunder Bay (TBRD-INMU-CH-6.18), and Lake Michigan, Milwaukee Bay (LMMB-01-INMU-6.18). While most PPCP compounds quantified in this study were detected at relatively low concentrations (< 10 ng/g wet weight) in mussels from open-water (61%, 43/70) and developed sites (56%, 39/70), their detection as complex mixtures in this study might warrant additional assessment and prioritization, since information regarding their toxicity, fate, potential bioaccumulation, and endpoint is lacking and at times not fully understood. However, the potential adverse bio-effects and risks posed to aquatic organisms, directly associated with individual PPCPs quantified in dreissenid mussels, are beyond the scope of this study and are addressed in further detail in Fuller et al., (2023; in press).

## Conclusion

In this study, a broad suite of PPCPs were assessed and characterized to better understand the environmental occurrence, magnitude, and spatial distribution of these emerging contaminants in relation to site land-use categories and proximity to point source dischargers within the Great Lakes Basin inshore (harbors, rivers, embayment, tributaries) and offshore locations. Our results revealed PPCPs were detected at all Great Lakes sites, mainly as complex mixtures, with 4 to 28 compounds detected at one or more mussel sampling locations. This emphasizes the need for continued monitoring, assessment and prioritization of these emerging contaminants, since information regarding their toxicity, fate, potential bioaccumulation, and endpoint is lacking, and at times not fully understood. Overall, several compounds comprising vasodilators, anti-inflammatory, antidepressants, X-ray contrast media, stimulants, antihypertensives, and chemotherapy classes of pharmaceuticals were consistently detected across sampling events, land-use and site discharge types. Results from the unsupervised RF classification identified patterns that were meaningful in assessing sites with the highest PPCP composition and exposures, as well as land-use categories with predominant mixtures. Significant differences between dominant site land-use categories, site discharge types, and PPCP contaminants measured in this study were detected. Overall, PPCP frequency was higher at sites sampled in proximity to WWTPs, confirming the ubiquity of these contaminants in wastewater treatment processes. Additionally, the findings in this study further revealed PPCP composition and loading was highest at non-WWTP sites, compared to other discharge types, thus confirming the importance of non-point sources as important pathways of PPCPs detected within the Great Lakes Basin. Equally significant, strong correlations were observed between several classes of pharmaceuticals and site dominant land-use categories, site population estimates, point source, and wastewater parameters. The findings of this study will help in identifying and contextualizing the relationship between detected contaminants, various point source and land-use gradients, and their likely “sinks” and “hot spots” along the Great Lakes Basin coastal zone and adjacent watersheds. These efforts will further aid in prioritizing MWP sampling locations and contaminants to be monitored, thus making the most efficient use of resources and funds, while identifying best management practices (BMPs) that can support measures in reducing and abating the continued occurrence of PPCPs in stressed aquatic environments.

### Supplementary Information

Below is the link to the electronic supplementary material.

Supplementary file1 (DOCX 1543 KB)

Supplementary file2 (XLSX 145 KB)

## Data Availability

All associated data presented in this manuscript is available within the Supplementary Data file.
