# Supplementary material for: An assessment and characterization of pharmaceuticals and personal care products (PPCPs) within the Great Lakes Basin: Mussel Watch Program (2013–2018)
Source: Environ Monit Assess. 2024 Mar 5;196(4):345. doi: 10.1007/s10661-023-12119-3 (PMC10912168; doi:10.1007/s10661-023-12119-3)
Supplement: Supplementary file 1 — Supplementary file1 (DOCX 1543 KB) [file 10661_2023_12119_MOESM1_ESM.docx]

**Supplementary Information**

**Title:** An Assessment and Characterization of Pharmaceuticals and Personal Care Products (PPCPs) within the Great Lakes Basin: Mussel Watch Program (2013 - 2018).

**Authors:** Edwards, M, A. ^1^*, Kimbrough, K.^1^, Fuller, N.^2,^ Davenport, E.^1^, Rider, M.^2^, Freitag, A.^1^, Regan, S.^2^, Leight, A, K.^1^, Burkart, H.^2^, Jacobs, A.^2^, and Johnson, E.^1^

^1^Monitoring and Assessment Branch, NOAA/NOS/NCCOS, 1305 East/West Highway, Silver Spring, MD 20910, USAFig

^2^CSS-Inc., Under NOAA National Centers for Coastal Ocean Science Contract No. EA133C17BA0062 & EA133C17BA0049, Fairfax, VA, USA

* Corresponding Author: Michael A. Edwards

Tel.: +1 240 533 0374

Fax: +1 301 713 4384

E-mail address: Michael.Edwards@noaa.gov


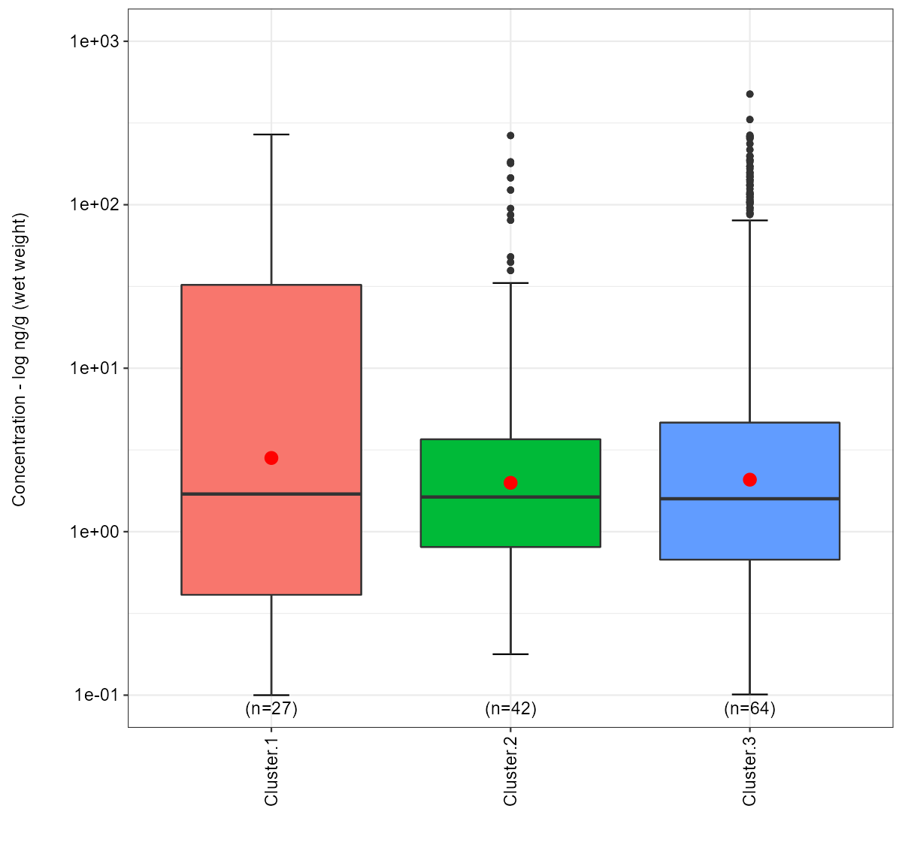


Fig. S1. Box and whisker plots depicting clusters (clusters 1-3) from the unsupervised RF model classification results and PPCP concentration [log ng/g (wet weight)] profile, quantified in dreissenid mussels during 2013-2018. Red dots within each plot represent mean values, horizontal lines indicate median concentration, while outliers are depicted as individual points. Numbers in parentheses above the x-axes indicate number of PPCP compounds detected within each RF cluster. Additional information is provided in Table S3 (**Supplementary Information**).


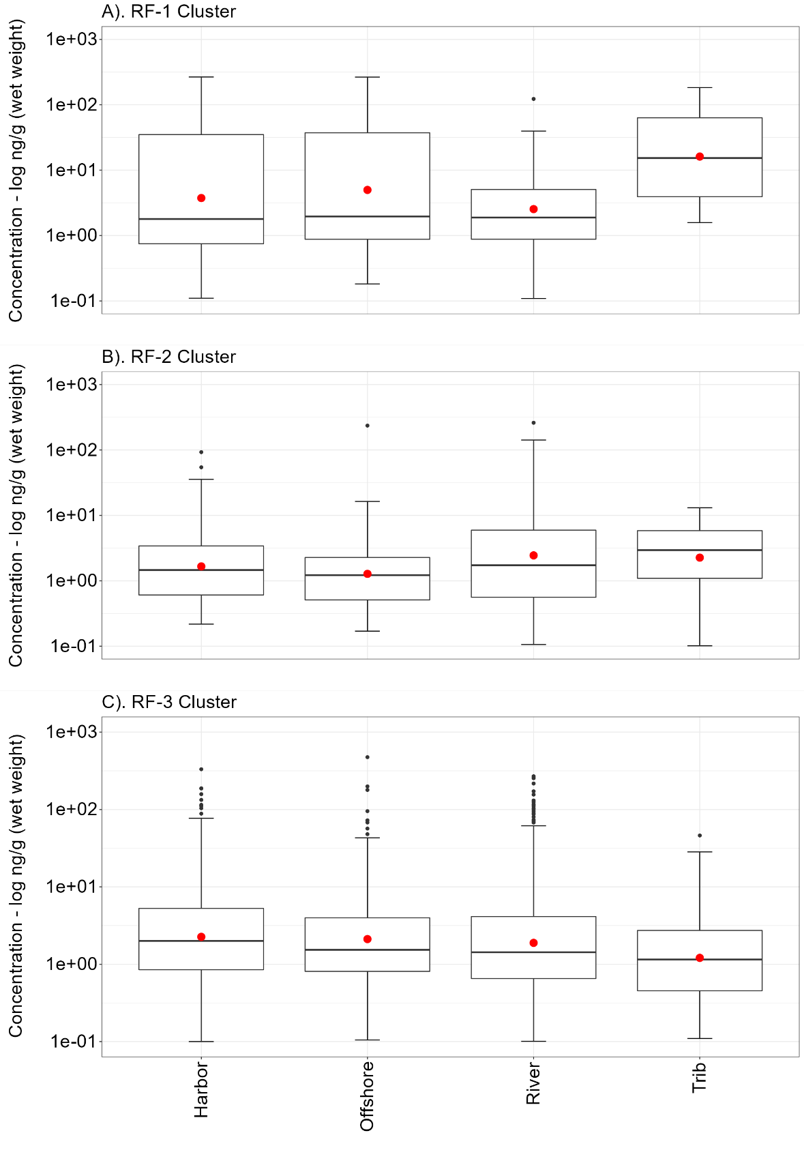


Fig. S2. Box and whisker plots showing variation in the concentration profile [log ng/g (wet weight)] for PPCP compounds observed in RF clusters 1-3 at nearshore, offshore, river and tributary (trib) sites. PPCP contaminant levels are based on concentrations within each RF cluster nearshore, offshore, river and tributary sites. Red dots within each plot represent mean values, horizontal lines indicate median concentrations, while outliers are depicted as individual points.


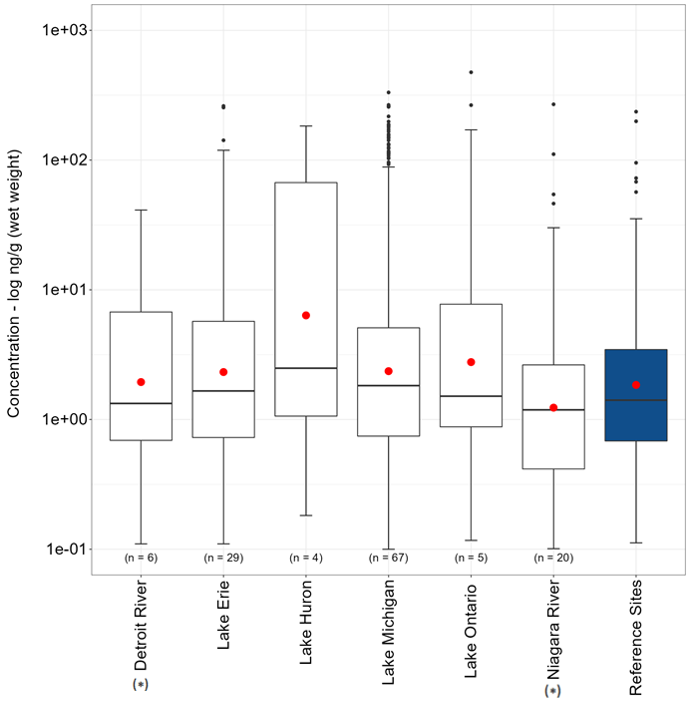


Fig. S3. Box and whisker plots showing PPCP concentration profile [log ng/g (wet weight)] detected in dreissenid mussels sampled at sites in Lake Michigan, Lake Huron, Lake Erie, Lake Ontario, Detroit and Niagara River connecting channels (*), and designated MW reference sampling locations between 2013 and 2018. Red dots within each plot represent mean values, horizontal lines indicate median concentrations, outliers are depicted as individual points, while *n* refers to the number of sites sampled within each Great Lakes and connecting channel. Additional information on the Great Lakes MWP designated reference sites is provided in Table 3 and Table S1.


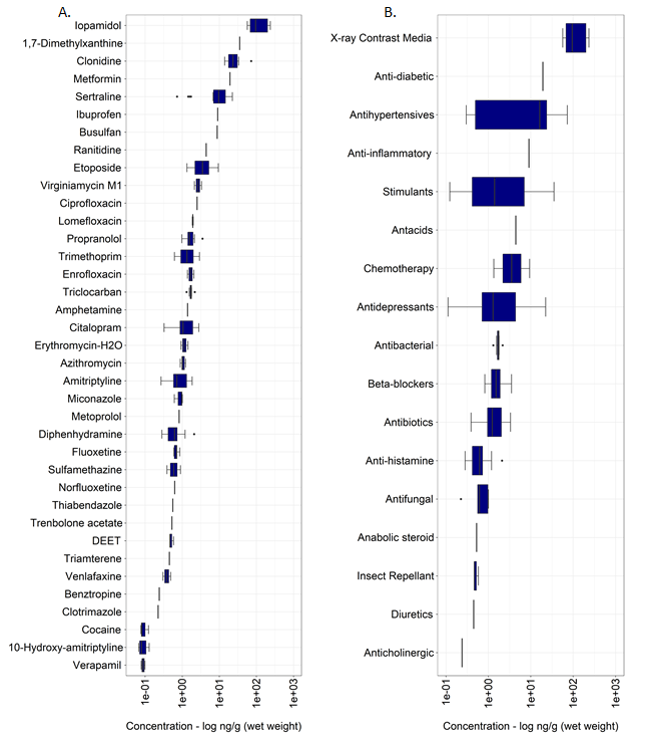


Fig. S4. Box and whisker plots depicting PPCP concentrations [log ng/g (wet weight)] detected in dreissenid mussels sampled at designated Great Lakes mussel watch (MW) reference sites during 2013-18. Figure (A) depicts PPCP concentrations summarized by compounds in descending order based on highest to lowest mean concentration value, while figure (B) groups the same compounds and depicts PPCP concentration, summarized by compound class in descending order based on highest to lowest mean concentration. The x axis (log scaled) represents several orders of magnitude difference in PPCP compounds and compound class concentrations quantified in dreissenid mussel tissue samples. Additional information is provided in Table S6.


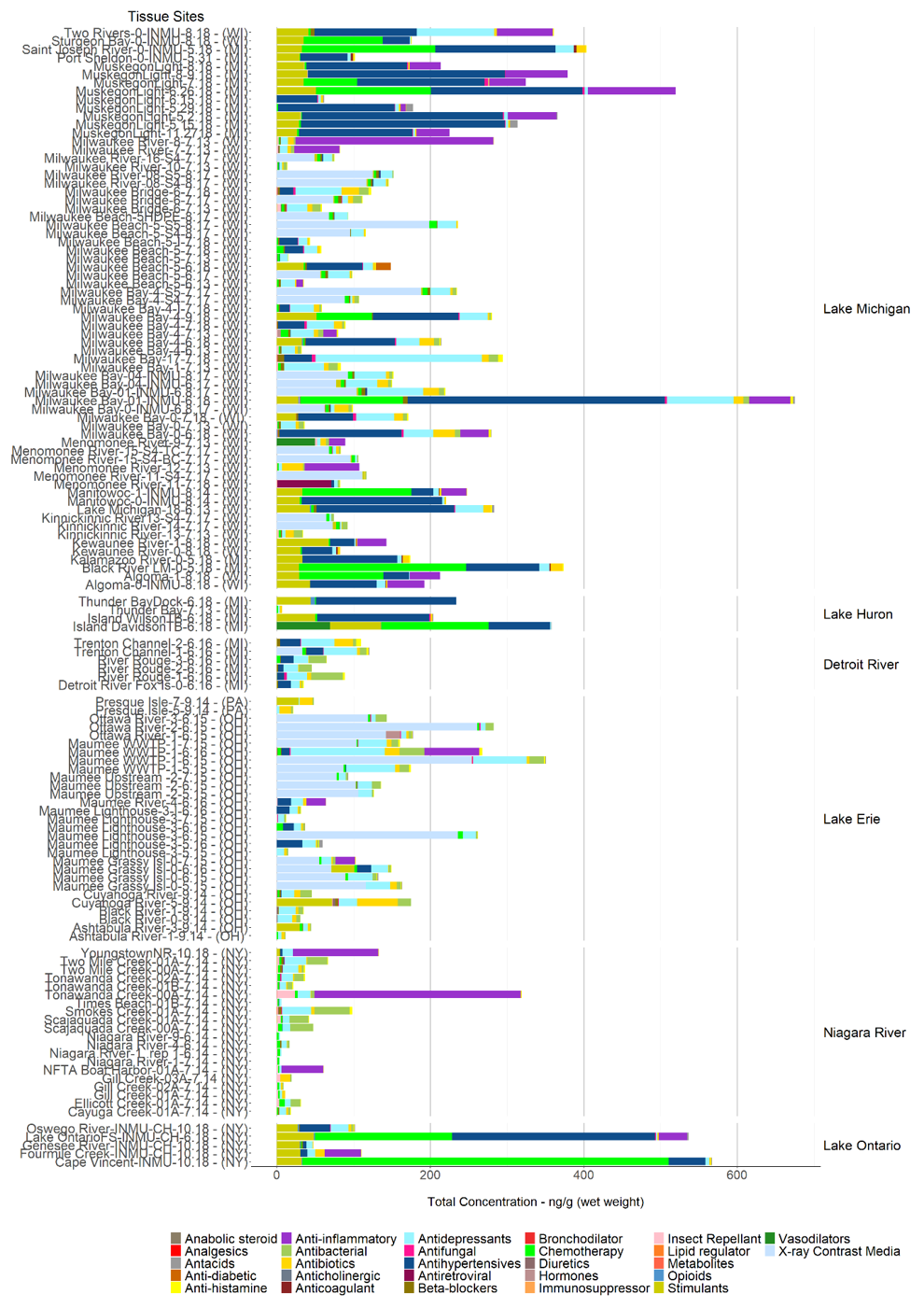


Fig. S5.  Bar plot depicting spatial distribution and summed PPCP concentration [ng/g (wet weight)] profile, computed by compound class measured in dreissenid mussels at each Great Lakes MWP study site between 2013 and 2018. Individual sites are listed by their general location (associated river/lake region), month and year sampled, and state which corresponds with mussel study sites provided in Table S1 (Supplementary Information). Additional information on MWP study sites summed PPCP concentration profile is provided in Table S7.


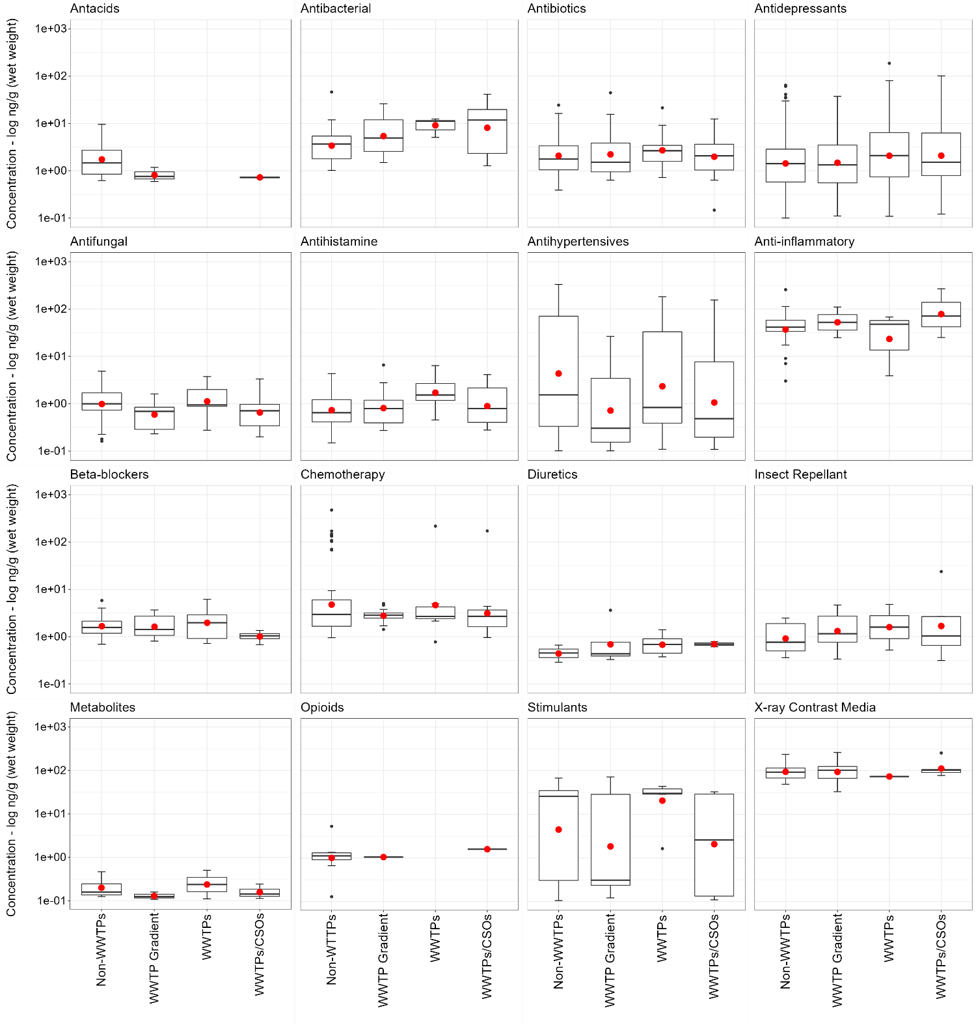


Fig. S6.  Box and whisker plots showing overall variation in PPCP compound class concentration [log ng/g (wet weight)] detected in dreissenid mussels sampled at major discharge-types in the Great Lakes. PPCP contaminant levels are based on cumulative concentrations within each compound group. Only PPCP compound groups detected at ten (10) or more sites are summarized in the above plots. Red dots within each plot represent mean values, horizontal lines indicate median concentration, while outliers are depicted as individual points. Abbreviations; WWTP/CSOs: sites sampled in proximity to wastewater treatment plants (WWTPs) and combined sewer overflow (CSOs) point source; WWTPs: sites sampled in proximity to wastewater treatment plants (WWTPs) only; WWTP Gradient: sites sampled downstream and along gradients of wastewater discharge; Non-WWTPs: sites sampled with neither WWTP nor CSO point source discharge. Additional information is provided in Table S8.


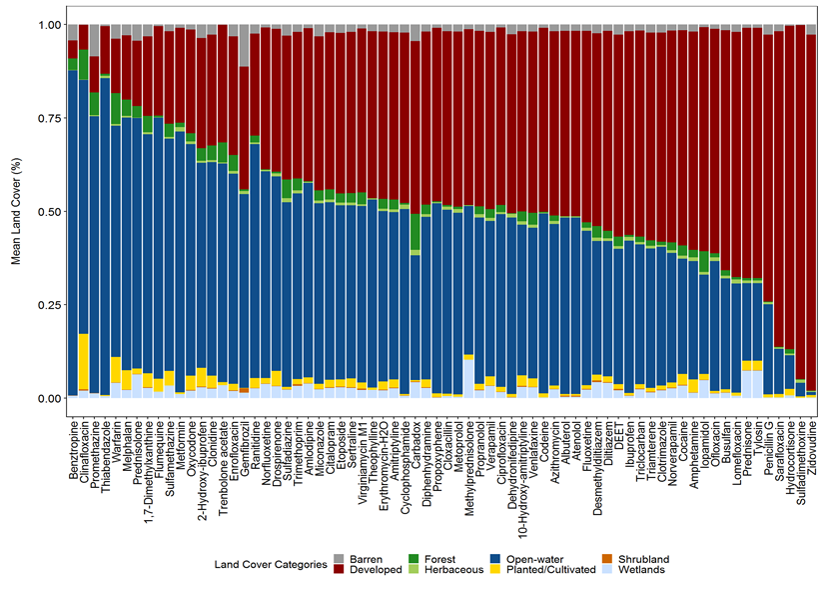


Fig. S7. Barplot depicting PPCP association and distribution within individual land-use category. PPCP distribution within each land-use category is presented in order from greatest (left) to least open-water category.


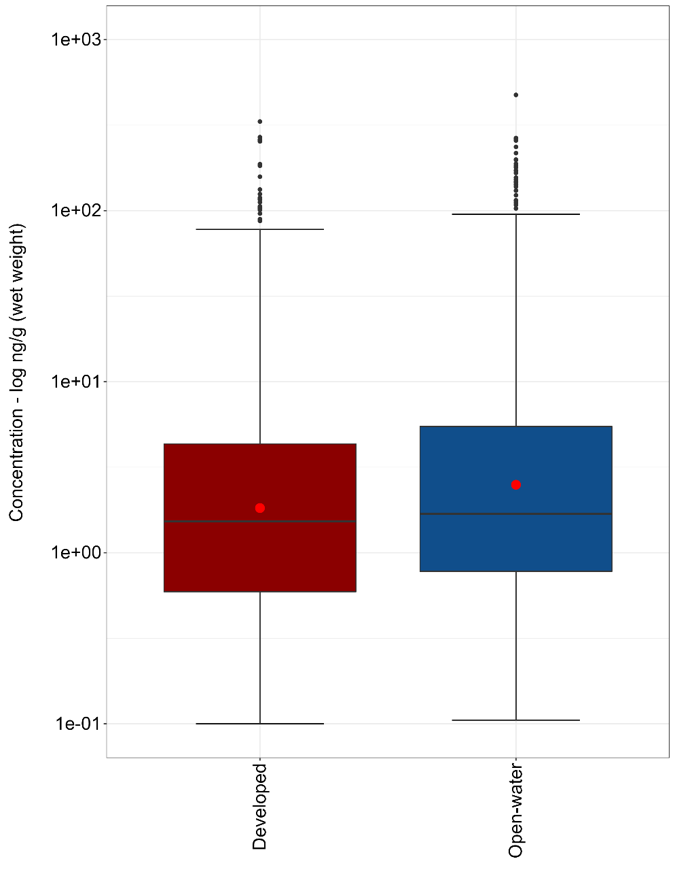


Fig. S8. Box and whisker plots depicting variation in PPCP concentration [log ng/g (wet weight)] detected in mussel tissue sampled at developed (> 45% land cover), and open-water sites. Red dots within each plot represent mean values, horizontal lines indicate median concentration, while outliers are depicted as individual points. Additional information is provided in Table S11.


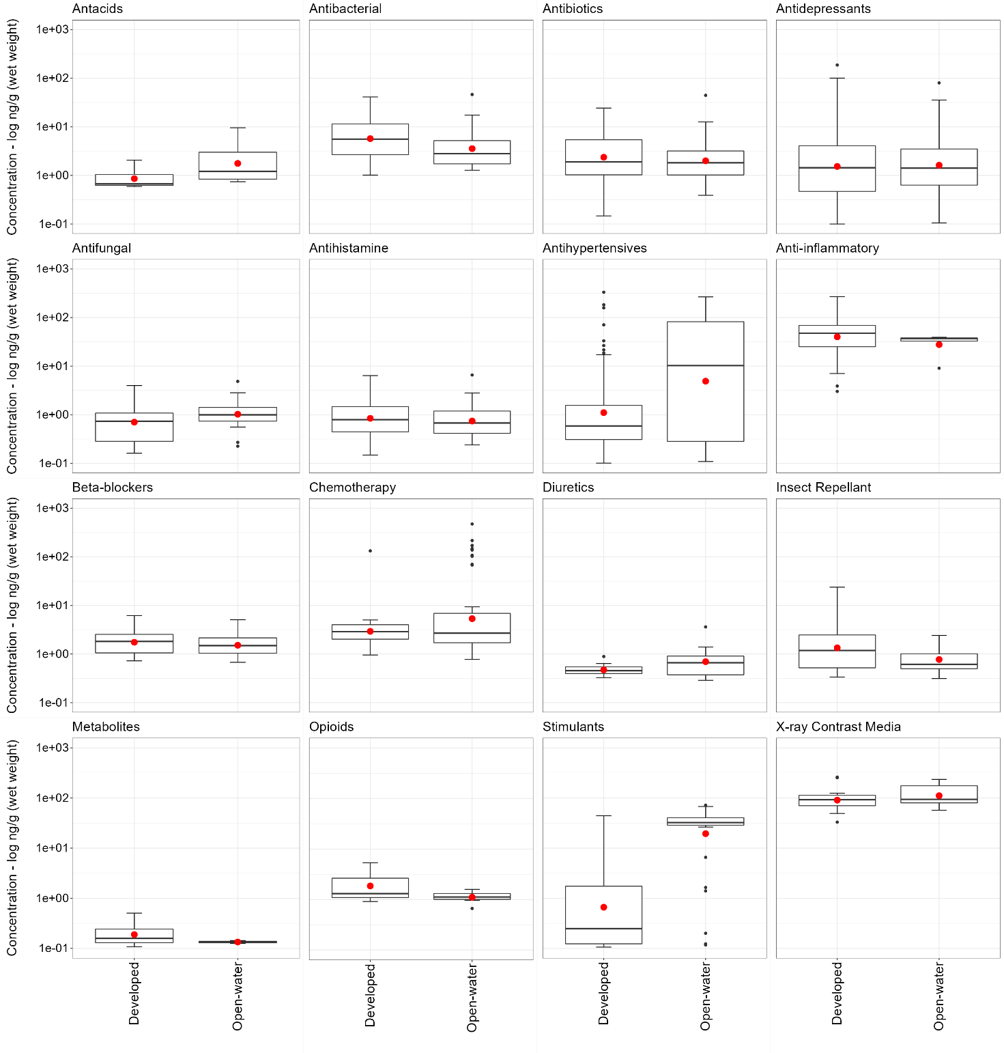
Fig. S9.  Box and whisker plots depicting overall variation in PPCP compound groups concentration [log ng/g (wet weight)], detected in dreissenid mussels sampled at developed and open-water sites. PPCP contaminant levels are based on cumulative concentrations within each compound group. Red dots within each plot represent mean values, horizontal lines indicate median concentration, while outliers are depicted as individual points. Only PPCP compound groups detected at ten (10) or more sites are summarized in the above plots.
